# Refractory Nitride, Resilient PCM: Titanium Nitride/RT70 HC Nanocomposites for Medium-Temperature Thermal Energy Storage and Management

**DOI:** 10.3390/molecules31152572

**Published:** 2026-07-23

**Authors:** Elshan Sefidgar Shahanaghi, Yasin Varol, Ezgi Gürgenç, Şafak Melih Şenocak, Ayşe Biçer, Turan Gürgenç

**Affiliations:** 1 Department of Automotive Engineering, Faculty of Technology, Fırat University, 23119 Elazig, Türkiye; elshansefidgar1@gmail.com (E.S.S.); yvarol@firat.edu.tr (Y.V.); 2 Graduate School of Natural and Applied Sciences (Automotive Engineering), Fırat University, 23119 Elazig, Türkiye; 3 Department of Mechanical Engineering, Faculty of Technology, Fırat University, 23119 Elazig, Türkiye; egurgenc@firat.edu.tr; 4 Department of Hybrid and Electric Vehicles Technology, Osmaniye Korkut Ata University, 80000 Osmaniye, Türkiye; safakmelihsenocak@osmaniye.edu.tr; 5 Department of Bioengineering, Malatya Turgut Özal University, 44210 Malatya, Türkiye; ayse.bicer@ozal.edu.tr

**Keywords:** titanium nitride nanocomposite, TiN, phase change material, thermal reliability, high thermal stability

## Abstract

This study tailors titanium nitride (TiN)-reinforced RT70 HC nanocomposite phase change materials (PCMs) for medium-temperature thermal energy storage. TiN nanoparticles were incorporated into commercial RT70 HC at 0.1–2.0 wt.% by a two-stage method combining sodium dodecyl sulfate, magnetic stirring, and ultrasonication, and characterized by FT-IR, XRD, SEM-EDX, elemental mapping, DSC, thermal conductivity, Cp, TGA, and 1000-cycle tests. FT-IR and XRD confirmed the physical integration of TiN into RT70 HC without new chemical bonds or secondary phases, and SEM-EDX showed a concentration-dependent dispersion. The phase change temperatures were largely preserved. The latent heat varied non-monotonically with TiN content, increasing at low loadings (0.1–0.5 wt.%) and decreasing at higher loadings. Because each composition was prepared as a single batch and measured on small specimens, the low-loading latent-heat increase (up to about 9%) is indicative rather than statistically proven and may fall within the subsampling variance. The 0.5 wt.% sample reached the highest values of 306/296 J/g in the first cycle and 286/268 J/g after 1000 cycles. The thermal conductivity increased with TiN content, reaching a maximum enhancement of about 24.09% in the liquid phase (0.1785 to 0.2215 W/(m·K) at 80 °C) and 35.7% in the solid phase at 2.0 wt.%, whereas the specific heat capacity was lower at higher loadings, an indicative trade-off given the single-run measurement uncertainty. TGA showed degradation onset temperatures above 240 °C, a wide safety margin relative to the ~72 °C working range. Overall, the 0.1–0.5 wt.% formulations offered the most balanced thermal performance for medium-temperature applications.

## 1. Introduction

The continuous increase in global energy consumption, the gradual depletion of conventional fossil energy sources, and the increasingly evident environmental problems caused by greenhouse gas emissions have substantially increased the need for clean energy production, more efficient energy utilization, and reliable energy storage technologies [1,2,3]. In particular, the temporal mismatch between the availability of intermittent renewable energy sources such as solar and wind and the actual consumption profile remains one of the most critical engineering problems today. To bridge this gap, thermal energy storage (TES) systems have emerged as an indispensable component for the effective management of intermittent thermal sources [2,4,5]. Thermal energy can be stored through three fundamental mechanisms: chemical heat storage based on reversible bond-breaking and bond-forming reactions, sensible heat storage associated with the temperature change of a medium, and latent heat thermal energy storage (LHTES) achieved through the solid–liquid phase transformation of a working material [1,6,7]. Among these mechanisms, LHTES is regarded as the most attractive approach owing to its high storage density, nearly isothermal operating behavior, and ability to release a large amount of energy over a narrow temperature range [1,3,4,7].

The functional medium of LHTES systems is provided by phase change materials (PCMs), which can store and release a substantial amount of latent heat during melting and solidification cycles [7,8]. Owing to these distinctive features, PCMs have attracted considerable interest across a wide range of low- and medium-temperature applications such as solar collectors, photovoltaic/thermal (PV/T) systems, building thermal regulation, solar stills and solar-assisted desalination, food packaging, textile thermo-regulation, lithium-ion battery thermal management, and electronic cooling [2,4,7,9]. PCMs are generally classified into three main groups: organic (paraffins, fatty acids, sugar alcohols, esters), inorganic (salt hydrates, metallic alloys), and eutectic compounds [3,8]. Among these materials, paraffin-based organic PCMs are particularly attractive for low- and medium-temperature TES applications because of their high latent heat density, narrow phase change range, negligible supercooling behavior, chemical stability, non-corrosiveness, non-toxicity, and competitive cost [4,6]. Nevertheless, organic PCMs have two well-documented major drawbacks that severely limit their practical use: their intrinsically low thermal conductivity, which prolongs the thermal charging/discharging times and limits the heat exchange between the PCM and the surrounding heat transfer fluid, and their tendency to leak in the liquid phase, which adversely affects long-term structural stability [6,9,10,11].

Various strategies have been developed in the literature to overcome these limitations. These include the integration of metallic fins and embedded heat pipes [9,11], micro- and macro-encapsulation [12], shape stabilization within supporting frameworks such as expanded graphite, expanded perlite, exfoliated hexagonal boron nitride (h-BN) nanosheets, and biomass-derived porous carbons [6,10], and the direct dispersion of conductive nano-additives within the PCM matrix [5,7]. Among these approaches, the addition of nanoparticles to PCMs, thereby obtaining the systems referred to as nano-enhanced PCMs (NePCMs), has emerged as one of the most economical, scalable, and versatile techniques because of its ease of preparation, compositional tunability, and ability to effectively modify the thermophysical behavior of the base PCM [5,7]. The type, size, morphology, surface chemistry, concentration, and dispersion quality of the added nanoparticles jointly determine the resulting melting/solidification kinetics, thermal conductivity, specific heat capacity, latent heat retention, and cycling stability [5,7,13]. In this context, carbon-based additives (graphene, graphene oxide, multi-walled carbon nanotubes, expanded graphite), metallic nanoparticles (Cu, Ag, Ni), and ceramic oxides (Al_2_O_3_, TiO_2_, CuO, SiO_2_, Fe_3_O_4_) have been widely investigated [7,9,14]. However, numerous studies have shown that excessive nanoparticle addition can have an adverse effect: although the thermal conductivity continues to increase gradually, the latent heat capacity decreases markedly because of the reduction in the active PCM fraction, the partial immobilization of PCM chains at the particle/matrix interfaces, and the increasing tendency toward agglomeration [5,7]. For this reason, optimizing the additive concentration has become one of the most critical engineering problems in the design of high-performance NePCMs.

Within the broad family of nanostructured additives, transition-metal nitrides, and titanium nitride (TiN) in particular, have recently begun to attract attention as a unique and multifunctional ceramic additive phase [12,15,16]. TiN simultaneously combines properties that rarely coexist in a single phase, such as high intrinsic thermal conductivity, ceramic-like chemical and thermal stability extending up to high temperatures, high mechanical hardness, oxidation resistance, and metal-like free-electron behavior that gives rise to a pronounced localized surface plasmon resonance (LSPR) in the visible and near-infrared regions [12,15,16,17]. In the solar-assisted TES systems reported to date, this property allows TiN to act as both a broadband light absorber and a thermally conductive filler, combining photothermal conversion with heat dissipation within the PCM matrix [12,16]. In recent studies, the thermal conductivity of pentaerythritol-based solid–solid PCMs enriched with 0.2 wt.% TiN was reported to increase by more than 100%, the photothermal conversion efficiency approached 91%, and more than 96% of the original energy storage density was retained [16]. Similarly, TiN/CNT hybrid layers coated on paraffin/TiO_2_ microcapsules were shown to enhance the effective thermal conductivity by approximately 151.5% and to cause only a limited latent heat loss even after long-term thermal cycling [12]. These findings indicate that TiN is a highly promising yet still markedly underexplored additive phase for organic PCM-based composite systems.

Recent work by the present group has shown that ceramic nanofillers can substantially improve the thermophysical performance of commercial RT-series paraffins. Hafnium carbide (HfC) dispersed in RT64 HC at 0.5–2.0 wt.% by a surfactant-assisted stirring and ultrasonication route increased the thermal conductivity from 0.21 to 0.367 W/(m·K) in the solid phase (+74.76%) and from 0.177 to 0.235 W/(m·K) in the liquid phase (+32.76%) at 2.0 wt.%, while improving the specific heat capacity and preserving the cycling stability [18]. In a related study, cubic boron nitride (cBN) in RT54 HC raised the solid- and liquid-phase thermal conductivities by 59.2% and 27.0%, respectively, at 2.0 wt.%, together with an increased degradation onset temperature [19]. These studies indicate that the conductivity gain, the accompanying change in the latent and sensible heat, and the cycling behaviour depend strongly on the specific filler and base paraffin, so that each filler and PCM pair requires a dedicated, concentration-resolved evaluation rather than an extrapolation from other systems.

Despite this high potential, the literature on the enrichment of commercial paraffin PCMs operating in the technologically critical 60–80 °C working window with TiN remains rather limited. The few available studies have focused either on solid–solid PCMs such as pentaerythritol [16] or on microencapsulated paraffin systems [12], whereas the direct dispersion of TiN nanoparticles into commercial RT-series paraffin PCMs has not yet been systematically investigated. Among commercial PCMs, RT70 HC is a particularly attractive candidate for medium-temperature applications: with a peak melting temperature around 70 °C and a high volumetric latent heat capacity, it shows a high degree of compatibility with the operating ranges of solar stills, solar-assisted desalination units, domestic hot-water systems, medium-temperature waste-heat recovery applications, and PV/T hybrid collectors [11,12,14]. This 60–80 °C window is technologically important because it coincides with the delivery temperatures of domestic hot-water systems, the operating range of many flat-plate and PV/T solar collectors, and low-grade industrial waste-heat streams, for which few commercial PCMs combine an adequate latent heat with a sufficient thermal conductivity. Transient numerical simulations have shown, for example, that placing RT70 HC beneath the basin of a solar still increased the overall efficiency from 55.78% to 66.70% and raised the daily cumulative water productivity from 4.53 kg/m^2^ to 5.42 kg/m^2^ [11]. Additively manufactured aluminum 3D periodic structures, on the other hand, were shown to markedly accelerate the melting and solidification behavior of RT70 within latent heat storage units, confirming that RT70 exhibits a high sensitivity to thermal conductivity enhancement strategies [20]. Nevertheless, the intrinsically low thermal conductivity of RT70 HC (~0.2 W/(m·K)) continues to limit its full-scale use, and no study systematically examining the structure–property relationship of RT70 HC-based composites enriched with TiN nanoparticles has been published to date. Two specific gaps therefore remain. First, the direct dispersion of TiN in a commercial RT-series paraffin operating in the 60–80 °C window has not been reported. Second, the concentration-dependent trade-off between the thermal-conductivity gain and the latent-heat retention, together with the long-term reliability over up to 1000 melting-solidification cycles, has not been quantified for TiN-based paraffin composites. The present study addresses both gaps directly.

To fill this gap, the present study reports the development and comprehensive characterization of a novel series of RT70 HC_TiN nanocomposite PCMs containing 0.1, 0.5, 1.0, 1.5, and 2.0 wt.% TiN nanoparticles, prepared by a melt-blending method comprising the thermal melting of the PCM followed by sequential magnetic stirring, bath ultrasonication, and probe ultrasonication steps. In the present research, a low TiN concentration range (≤2 wt.%) was deliberately selected. This choice aims to maximize the thermal conductivity gain while minimizing the latent heat capacity loss associated with the reduction in the active PCM fraction. The crystal structure, chemical bonding characteristics, surface morphology, elemental distribution, melting-solidification behavior, specific heat capacity, thermal conductivity, thermal stability, and cycling reliability of the prepared composites were examined by XRD, FT-IR, FE-SEM/EDX and elemental mapping, DSC, TGA, and thermal conductivity measurements, and these analyses were supported by accelerated melting-solidification cycling tests applied up to 500 and 1000 cycles. To the best of our knowledge, the present study is among the first to directly disperse TiN nanoparticles into a commercial RT-series paraffin PCM, to systematically investigate the concentration-dependent thermophysical and structural behavior of RT70 HC at low TiN loadings, and to evaluate the cycling reliability of TiN-modified PCMs under repeated operating conditions extending up to 1000 melting-solidification cycles. The results obtained are expected to contribute to the development of multifunctional, thermally stable, and cycling-durable nanocomposite PCMs for solar-assisted desalination, PV/T systems, solar-assisted domestic hot-water production, medium-temperature waste-heat recovery, and other thermally demanding energy storage applications. Although TiN is known to exhibit strong photothermal light absorption through its localized surface plasmon resonance, this property was not evaluated in the present study, and the photothermal characterization of the RT70 HC_TiN composites, for example under solar illumination, is therefore identified as a direction for future work.

## 2. Results and Discussion

### 2.1. Macroscopic Morphology

**Figure 1:**
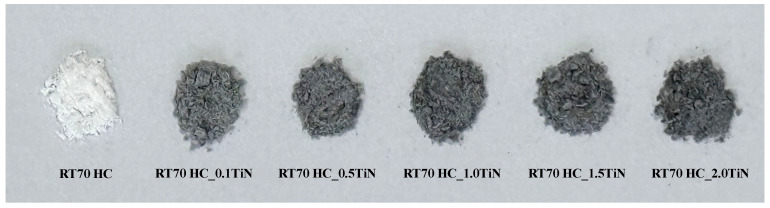
Macroscopic images of the TiN nanoparticles, pristine RT70 HC, and RT70 HC_TiN nanocomposite PCMs.

Figure 1 shows macroscopic images of the TiN nanoparticles, pristine RT70 HC, and the RT70 HC_TiN nanocomposite PCMs prepared at different nanoparticle concentrations. With increasing TiN content, the appearance darkened from the whitish tone of pristine RT70 HC toward grayish-black, consistent with the incorporation of the dark TiN phase into the matrix. No macroscopic phase separation, sedimentation, or large-scale agglomeration was observed at any additive ratio, which suggests that the melt-blending together with the SDS-assisted stirring and ultrasonication steps produced macroscopically homogeneous formulations. This macroscopic observation provides only a preliminary, qualitative indication of the dispersion state and is complemented by the SEM, EDX, and elemental-mapping analyses presented below.

### 2.2. FT-IR

**Figure 2:**
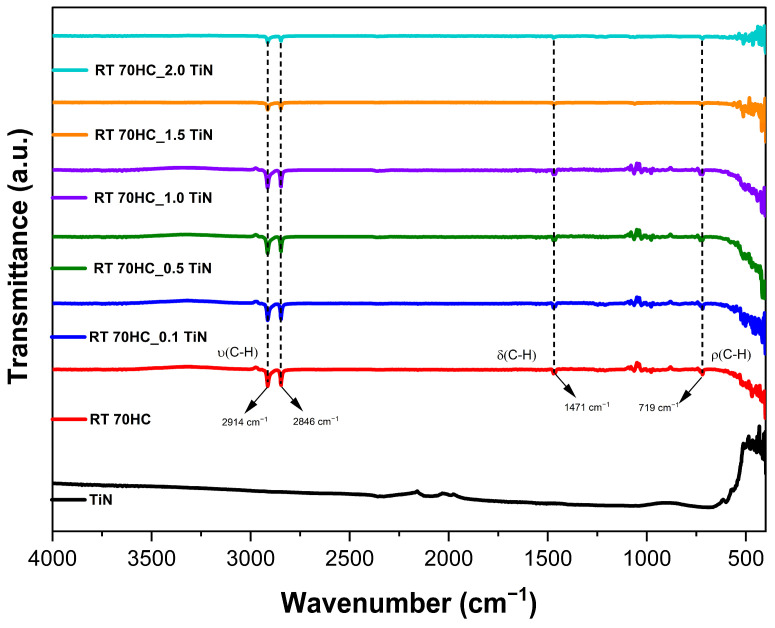
FT-IR spectra of the TiN nanoparticles, pristine RT70 HC, and RT70 HC_TiN nanocomposite PCMs before thermal cycling.

FT-IR analysis was performed to examine the chemical structure of the materials, to evaluate the compatibility between RT70 HC and TiN, and to clarify whether any chemical interaction or new bond formation occurred during the melt-blending preparation process. In addition, this analysis was used to confirm that the characteristic functional groups of the PCM are preserved and to evaluate the chemical stability of the system after the addition of TiN nanoparticles. The FT-IR spectra of the TiN nanoparticles, pristine RT70 HC, and RT70 HC_TiN nanocomposite PCMs are presented in Figure 2. The spectrum of the TiN nanoparticles did not exhibit any pronounced absorption features attributable to organic functional groups over the 4000–400 cm^−1^ region, whereas a broad absorption developing near the lower end of the spectrum, below approximately 500 cm^−1^, can be associated with the characteristic Ti-N lattice vibrations of titanium nitride [21,22]. This relatively featureless, organic-free profile is consistent with the inorganic nitride structure of the additive phase. The spectrum of pristine RT70 HC exhibits its main absorption bands at approximately 2914 cm^−1^, 2846 cm^−1^, 1471 cm^−1^, and 719 cm^−1^. The bands at 2914 and 2846 cm^−1^ are assigned to the asymmetric and symmetric aliphatic C-H stretching vibrations of the methyl and methylene groups in the paraffinic PCM structure [22]. The absorption band observed at 1471 cm^−1^ is associated with the bending (deformation) vibration of the -CH_3_ and -CH_2_ hydrocarbon groups, while the band at 719 cm^−1^ corresponds to the rocking vibration of the C-H bonds in the long-chain hydrocarbon structure [22,23]. These bands represent the fundamental fingerprint of the paraffin-based RT70 HC matrix. With the addition of TiN nanoparticles, the same characteristic RT70 HC bands were preserved in all nanocomposite PCM spectra. The peak intensities changed slightly with increasing TiN content, but the positions of the absorption bands did not show any significant shift compared with pristine RT70 HC. Moreover, no newly formed absorption band was detected in the nanocomposite spectra, and the characteristic RT70 HC functional groups remained visible after nanoparticle addition. The absence of new bands or notable peak shifts indicates that TiN incorporation did not induce any detectable chemical transformation in the RT70 HC structure [22,24]. In particular, no distinct absorption assignable to the sulfate group of SDS was resolved near 1220 cm^−1^, consistent with the low surfactant content. These results indicate that RT70 HC and TiN combined predominantly through physical interaction, without covalent bond formation between the components, during the melt-blending process. The preservation of the original RT70 HC absorption bands confirms the chemical compatibility between the PCM matrix and the TiN nanoparticles [21,24]. Therefore, the FT-IR results support that the TiN nanoparticles were incorporated into the RT70 HC matrix without disrupting the molecular structure of the PCM [22].

### 2.3. XRD

**Figure 3:**
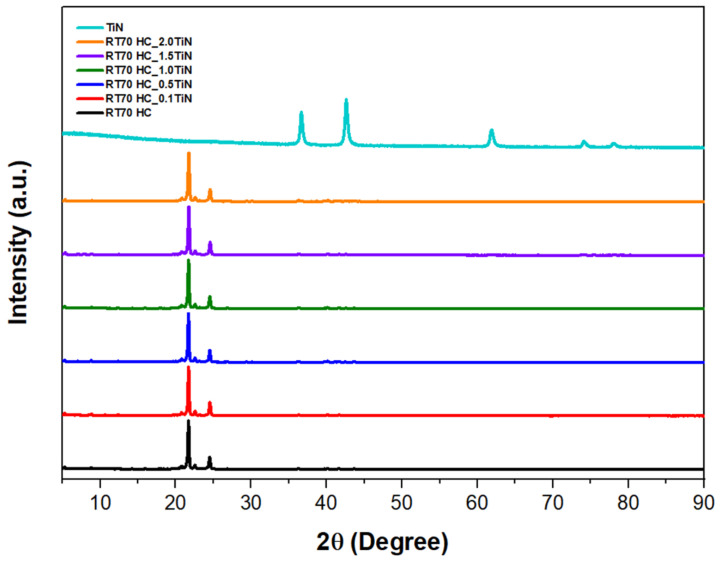
XRD patterns of the TiN nanoparticles, pristine RT70 HC, and RT70 HC_TiN nanocomposite PCMs.

The XRD patterns presented in Figure 3 reveal the crystalline characteristics of the TiN nanoparticles, pristine RT70 HC, and RT70 HC_TiN nanocomposite PCMs recorded over the 5–90° (2θ) range. The diffraction pattern of pristine RT70 HC exhibits two pronounced high-intensity reflections at approximately 21.5° and 23.8° (2θ), accompanied by much weaker reflections around 38–40° and 46° (2θ). These sharp and intense peaks are recognized as the characteristic reflections of the well-ordered orthorhombic subcell of the long-chain paraffinic structure of RT70 HC [25,26]. The high intensity and sharpness of these reflections confirm the highly crystalline structure of the pristine PCM, which is directly related to its high latent heat storage capability [26]. The diffraction pattern of the pristine TiN nanoparticles shows pronounced reflections at approximately 36.7°, 42.7°, 62°, 74°, and 78° (2θ), which correspond to the (111), (200), (220), (311), and (222) crystal planes of the face-centered cubic (fcc) rock-salt structure of titanium nitride [27,28]. The sharp and well-defined nature of these reflections indicates the high crystallinity and phase purity of the cubic TiN nanoparticles used as the additive phase [29]. In the nanocomposite PCMs, the characteristic RT70 HC peaks at approximately 21.5° and 23.8° were preserved in all samples, indicating that the fundamental crystal structure of the paraffinic PCM remained intact after the addition of TiN nanoparticles [30,31]. In addition to the dominant paraffinic reflections, the characteristic TiN diffraction peaks, particularly those at approximately 36.7° and 42.7°, gradually appeared in the nanocomposite patterns and became progressively more intense with increasing TiN content. At low additive ratios, especially in the RT70 HC_0.1TiN and RT70 HC_0.5TiN samples, the TiN reflections were very weak and almost indistinguishable, whereas they became visible at higher loadings such as 1.5 and 2.0 wt.%. This concentration-dependent behavior can be explained by the increasing contribution of the crystalline TiN phase within the composite and the simple superposition of the TiN reflections on the paraffinic diffraction background [32]. Importantly, no additional diffraction peak corresponding to a secondary phase, impurity, or newly formed crystalline compound was detected in the nanocomposite samples. This indicates that the prepared RT70 HC_TiN nanocomposites have a high phase purity and that the diffraction patterns represent only a physical superposition of two separate phases [22,33]. A degree-of-crystallinity analysis of the paraffin reflections (the integrated crystalline-peak area relative to the amorphous scattering in the 15–30° region) gave values of approximately 86–89% for all compositions, with no systematic increase at low TiN loadings, so the XRD-derived crystallinity does not by itself account for the latent-heat increase observed at 0.1–0.5 wt.% TiN (Section 2.5). Moreover, consistent with the FT-IR results, the absence of new crystalline phases confirms that no chemical reaction occurred between RT70 HC and TiN during the melt-blending preparation process. It can therefore be concluded that the TiN nanoparticles were physically incorporated into the PCM matrix, forming a structurally stable and phase-pure RT70 HC_TiN nanocomposite system [26].

### 2.4. SEM-EDX

**Figure 4:**
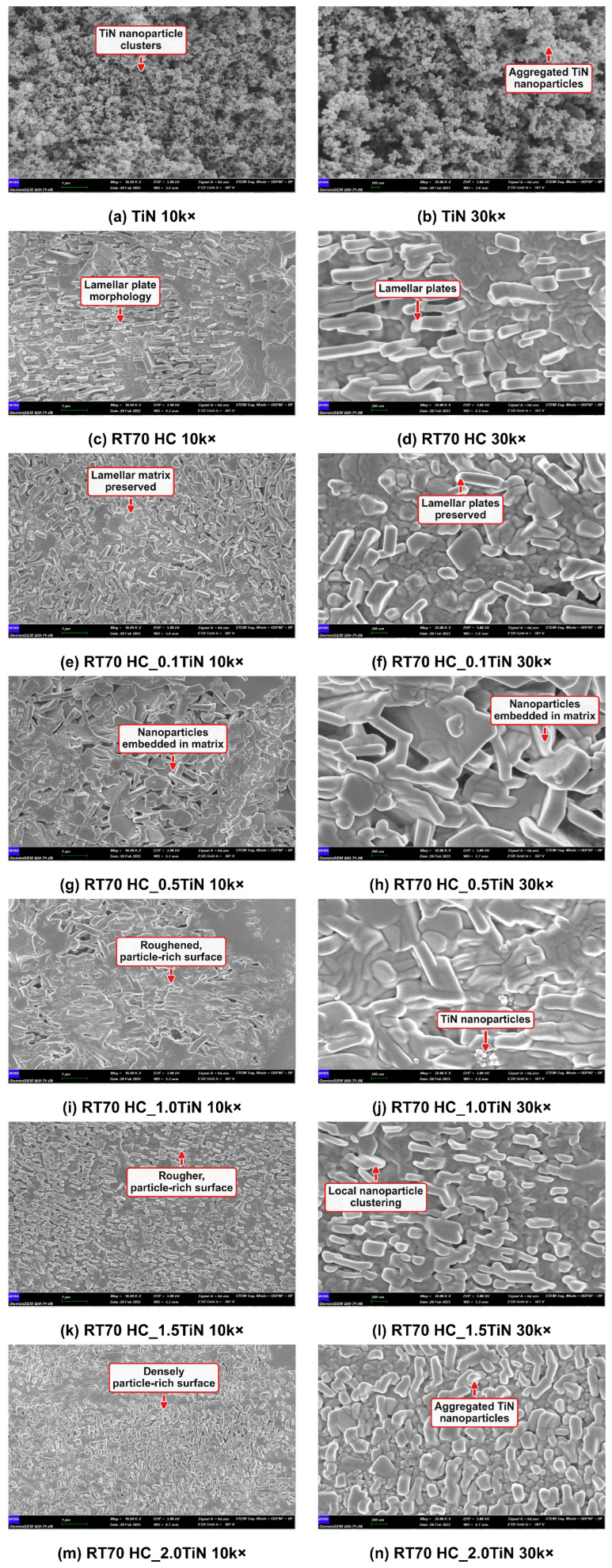
SEM images of (**a**) TiN at 10k×, (**b**) TiN at 30k×, (**c**) pristine RT70 HC at 10k×, (**d**) pristine RT70 HC at 30k×, (**e**) RT70 HC_0.1TiN at 10k×, (**f**) RT70 HC_0.1TiN at 30k×, (**g**) RT70 HC_0.5TiN at 10k×, (**h**) RT70 HC_0.5TiN at 30k×, (**i**) RT70 HC_1.0TiN at 10k×, (**j**) RT70 HC_1.0TiN at 30k×, (**k**) RT70 HC_1.5TiN at 10k×, (**l**) RT70 HC_1.5TiN at 30k×, (**m**) RT70 HC_2.0TiN at 10k×, and (**n**) RT70 HC_2.0TiN at 30k×.

**Figure 5:**
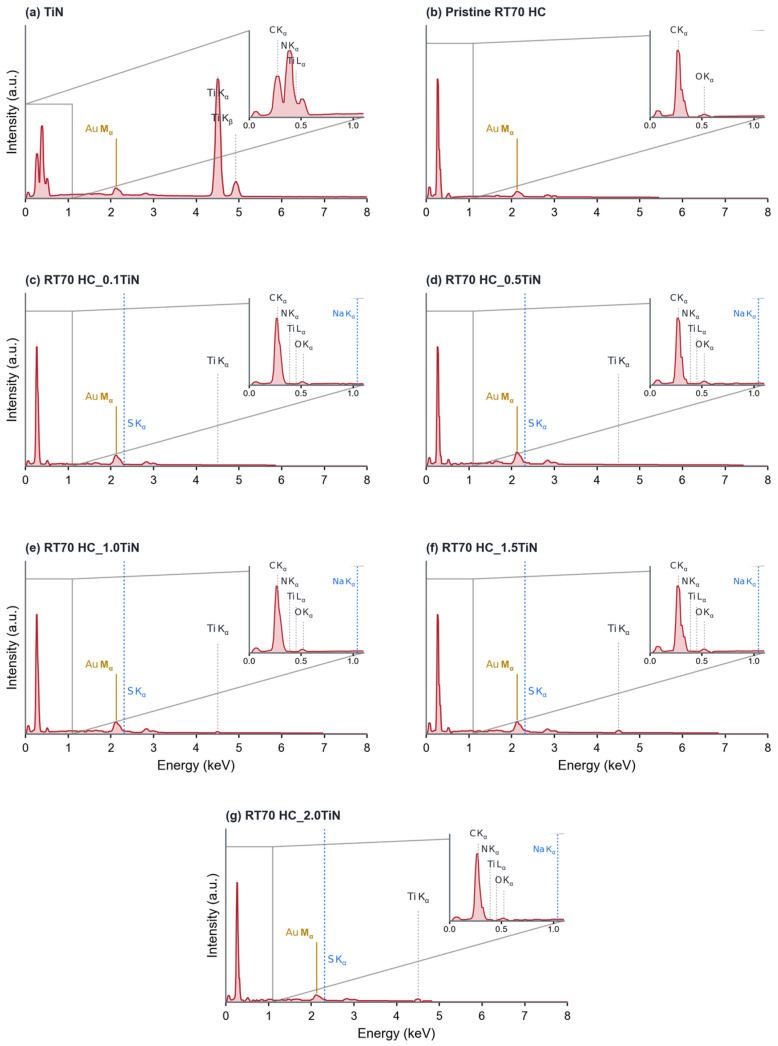
EDX spectra of (**a**) TiN nanoparticles, (**b**) pristine RT70 HC, (**c**) RT70 HC_0.1TiN, (**d**) RT70 HC_0.5TiN, (**e**) RT70 HC_1.0TiN, (**f**) RT70 HC_1.5TiN, and (**g**) RT70 HC_2.0TiN nanocomposite PCMs. In each panel, the Au Mα peak (gold) originates from the conductive gold coating, and the Na Kα and S Kα markers (blue) indicate the expected positions of the surfactant-related elements.

The surface morphologies and elemental compositions of the pristine RT70 HC, TiN nanoparticles, and RT70 HC_TiN nanocomposite PCMs were examined by SEM and EDX analyses. In this context, SEM images recorded at two magnifications (10k× and 30k×) are presented in Figure 4, while the EDX spectra are given in Figure 5. The SEM image of pristine RT70 HC shows a relatively smooth, continuous, and compact surface. At high magnification, the paraffinic matrix exhibits a well-developed lamellar plate-like crystalline morphology typical of long-chain organic PCMs [34,35]. In contrast, the TiN nanoparticles display a markedly different surface structure consisting of fine, irregular, and densely packed particle clusters that show a pronounced tendency to aggregate, reflecting the high surface energy of the nanoscale cubic nitride particles [36]. This morphological contrast demonstrates the structurally distinct nature of the organic paraffinic matrix and the inorganic nitride nanoparticles. The addition of TiN nanoparticles to RT70 HC caused gradual, concentration-dependent changes in the surface morphology of the nanocomposite PCMs. At low additive ratios, especially in the RT70 HC_0.1TiN and RT70 HC_0.5TiN samples, the continuous lamellar morphology of the PCM matrix was largely preserved, and the nanoparticles appeared embedded within and surrounded by the RT70 HC phase. This observation indicates that the molten RT70 HC effectively wetted and enveloped the TiN nanoparticles during the melt-blending process and that the nanoparticles were dispersed within the PCM matrix [5,34,37]. The two-stage SDS-assisted preparation route combining magnetic stirring, bath ultrasonication, and probe ultrasonication is thought to have provided closer contact between the nanoparticles and the PCM matrix, thereby contributing to the formation of a more homogeneous composite structure [5]. As the additive ratio increased, the surface morphology of the nanocomposites gradually became more irregular, rougher, and richer in particles. Especially in the samples containing 1.0, 1.5, and 2.0 wt.% TiN, the regions associated with the TiN phase became more pronounced, and the nanoparticles occupied a relatively larger area within the composite structure. This behavior is mainly attributed to the increasing volume fraction of the inorganic TiN phase in the PCM matrix and the higher probability of contact between the nanoparticles with increasing additive content [37,38]. Furthermore, although partial nanoparticle clustering and local agglomeration were observed in some regions at high additive ratios, the PCM matrix generally appeared to envelop and retain the nanoparticles within the composite structure. This indicates that RT70 HC physically immobilized the TiN nanoparticles and contributed to their stabilization within the nanocomposite matrix [5,39,40]. The EDX spectra, as shown in Figure 5, further support the incorporation of the TiN nanoparticles into the RT70 HC matrix. While pristine RT70 HC consists mainly of carbon together with a small amount of oxygen, the spectrum of the TiN nanoparticles shows nitrogen and titanium as the dominant elements. In the RT70 HC_TiN nanocomposite PCM spectra, carbon, oxygen, nitrogen, and titanium signals are observed together. While the carbon signal mainly originates from the RT70 HC matrix, the nitrogen and titanium peaks indicate the presence of the TiN nanoparticles within the composite structure [16]. The elemental composition values obtained from the EDX analysis are listed in Table 1. Because EDX is a semi-quantitative technique with limited accuracy for light elements such as carbon, nitrogen, and oxygen, the absolute C, N, and O values in Table 1 should be regarded as indicative rather than exact. In particular, the nitrogen contents lie close to the EDX detection limit for such a light element, so their non-monotonic variation (for example, 2.36, 0.89, and 4.60% for the 0.1, 0.5, and 1.0 wt.% samples) is not physically meaningful and is not over-interpreted here. Accordingly, the monotonic increase in the Ti content together with the residual-mass trend obtained from TGA provides the more reliable measure of the actual TiN incorporation. While pristine RT70 HC contains 92.77% C and 7.23% O, the pristine TiN nanoparticles contain 76.19% Ti and 23.81% N. In the nanocomposite PCMs, the Ti content generally increased with increasing additive ratio, rising from 0.12% for RT70 HC_0.1TiN to 0.43% for RT70 HC_0.5TiN, 0.76% for RT70 HC_1.0TiN, and 1.66% for RT70 HC_1.5TiN, reaching the highest value of 1.69% in the RT70 HC_2.0TiN sample. These results show that the TiN nanoparticles were incorporated into the RT70 HC matrix, generally in agreement with the intended additive ratios [16,38]. In contrast, the carbon content generally decreased as the TiN ratio increased, dropping from 92.77% in pristine RT70 HC to 85.09% in the 1.5 wt.% TiN sample, while the remaining nanocomposites exhibited carbon contents in the range of approximately 85–89%. This decrease is mainly attributed to the reduction in the relative amount of the organic PCM phase within the composite as the inorganic TiN fraction increased [39]. Some variations in the nitrogen and oxygen contents were also observed among the nanocomposite samples. The oxygen signal is thought to originate mainly from the oxygen-containing groups in the organic RT70 HC structure, while the small fluctuations in the measured nitrogen and oxygen contents can be associated with local structural differences within the analyzed EDX regions and the microscale non-uniform local distribution of the nanoparticles [24,37]. Apart from a low-intensity Au Mα peak at about 2.12 keV arising from the conductive gold coating, no elemental peak other than C, N, O, and Ti was detected in the EDX spectra. In particular, no sodium or sulfur signals from the SDS surfactant were resolved. This is expected because, even at the highest loading, the SDS corresponds to only about 0.3 wt.% Na and 0.4 wt.% S of the total, so the Na Kα (about 1.04 keV) and S Kα (about 2.31 keV) lines remain at or below the reliable EDX detection limit for these light elements in an organic matrix. This finding indicates that no elements other than those of the intended components were detected within the composite structure [16,36]. When evaluated together with the FT-IR and XRD results, the SEM and EDX analyses support that RT70 HC and the TiN nanoparticles combined physically without any chemical reaction and that the TiN nanoparticles were distributed throughout the PCM matrix. Overall, the results show that the TiN nanoparticles were increasingly incorporated into the RT70 HC matrix with increasing additive ratio and visibly affected the morphological and elemental properties of the composite structure.

**Figure 6:**
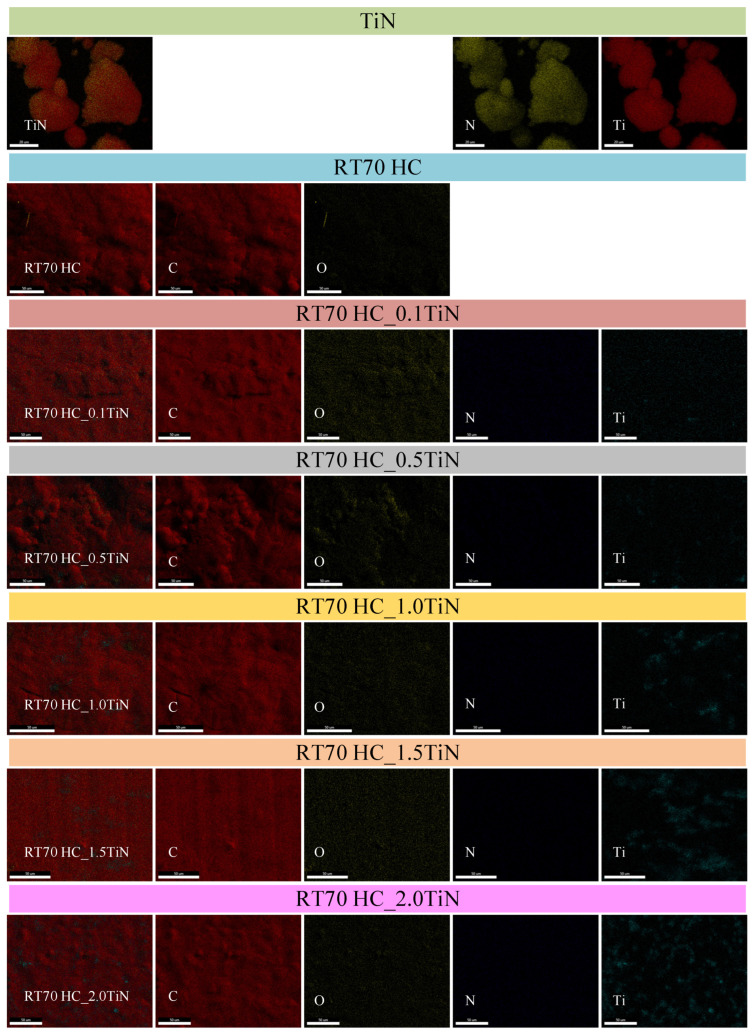
Elemental mapping images of the pristine RT70 HC, TiN nanoparticles, and RT70 HC_TiN nanocomposite PCMs showing the distribution of the C, O, N, and Ti elements within the composite structure. For the pure TiN reference, the C and O panels are left blank because titanium nitride contains no carbon or oxygen. The acquisition conditions (20 kV, EDAX TEAM system) were identical for all samples.

The elemental mapping images of the pristine RT70 HC, TiN nanoparticles, and RT70 HC_TiN nanocomposite PCMs are presented in Figure 6. In pristine RT70 HC, the C and O elements are distributed throughout the structure, while the N and Ti signals are observed in the TiN nanoparticles. In the TiN-added nanocomposites, the C and O signals represent the RT70 HC matrix, whereas the presence of N and Ti signals across the mapped regions is consistent with the incorporation of the TiN nanoparticles into the PCM matrix. As the additive ratio increased, the intensities of the Ti and N signals in the elemental maps became progressively more pronounced. This observation is consistent with the EDX composition results given in Table 1 and indicates that the titanium content within the nanocomposite structure increased with increasing TiN loading. Moreover, the absence of pronounced phase separation or large-scale local accumulation in the mapping images indicates that the TiN nanoparticles were generally distributed within the RT70 HC matrix [24,34,38]. At the lowest loading (0.1 wt.%, corresponding to only about 0.015 vol.% TiN), the additive concentration is below the effective sensitivity of elemental mapping, so the maps at this loading should not be read as direct evidence of a uniform dispersion. These results further support that the applied two-stage preparation method physically combined the PCM and the nanoparticles into an elementally compatible RT70 HC_TiN nanocomposite PCM structure.

### 2.5. DSC

**Figure 7:**
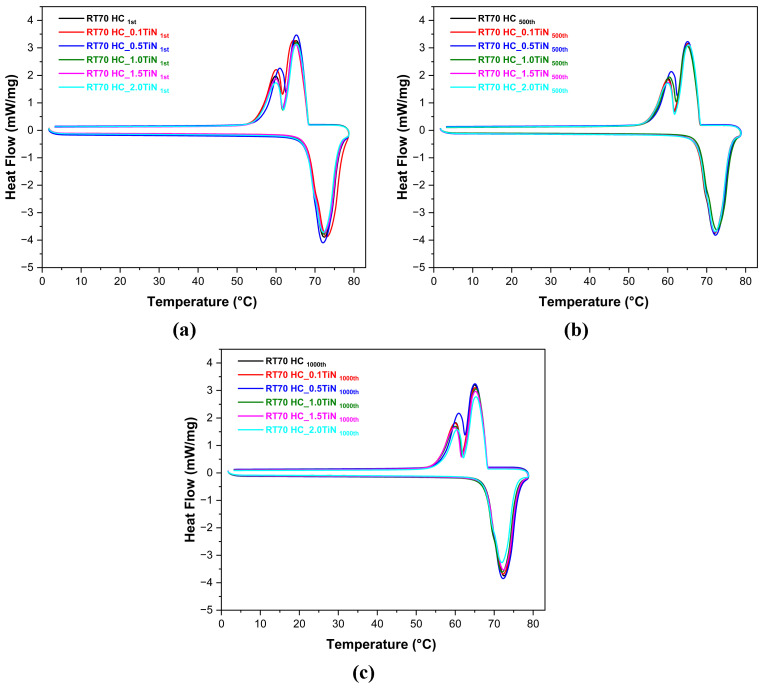
DSC melting and solidification curves of the pristine RT70 HC and RT70 HC_TiN nanocomposite PCMs obtained after the (**a**) 1st, (**b**) 500th, and (**c**) 1000th cycles.

The DSC melting and solidification curves of the pristine RT70 HC and RT70 HC_TiN nanocomposite PCMs after the 1st, 500th, and 1000th cycles are presented in Figure 7. The melting onset temperature (Tbm), melting peak temperature (Tpm), melting end temperature (Tfm), solidification onset temperature (Tbs), solidification peak temperature (Tps), and solidification end temperature (Tfs) values obtained from these curves are listed in Table 2 together with the ΔHm, ΔHs, ωm, ωs, and ΔT values defined in Section 3. Distinct endothermic and exothermic peaks were observed for the pristine RT70 HC and all RT70 HC_TiN nanocomposite PCMs. The preservation of the melting and solidification peaks at all additive ratios indicates that the TiN nanoparticles did not suppress or eliminate the fundamental phase change behavior of RT70 HC. This finding is consistent with the FT-IR and XRD results, which showed that no new chemical bonds or crystalline phases formed between RT70 HC and TiN and that the components combined mainly through physical interactions. The melting endotherms also show a minor pre-melting shoulder below the main peak, which is consistent with the solid–solid (rotator) transition of long-chain paraffins rather than with any TiN-related effect. For pristine RT70 HC, the Tbm, Tpm, and Tfm values in the 1st cycle were determined to be 68.7 °C, 72.4 °C, and 75.3 °C, respectively. The corresponding Tbs, Tps, and Tfs values were 67.4 °C, 65.2 °C, and 61.9 °C, respectively. The ΔHm and ΔHs values of pristine RT70 HC were obtained as 280 J/g and 275 J/g, respectively. The measured melting enthalpy (ΔHm = 280 J/g) differs by about 8% from the nominal heat storage capacity of 260 kJ/kg (i.e., 260 J/g) quoted in the RT70 HC datasheet. Because the datasheet value is a combined (latent plus sensible) quantity measured by the manufacturer over a defined temperature window, whereas the present ΔHm is the integral of the DSC melting peak, the two are not directly comparable, and this difference is noted here rather than treated as a validation [41]. The effect of TiN addition on the phase change temperatures was generally limited. In the 1st cycle, the Tpm values of the TiN-added samples ranged from 72.1 to 73.0 °C, while the Tps values ranged from 64.5 to 65.3 °C. The closeness of these values to those of pristine RT70 HC indicates that TiN addition did not significantly change the operating temperature range of RT70 HC and that the small fluctuations of about one-tenth of a degree observed in the peak temperatures are within the typical scatter reported for nanoparticle-containing composites and are not attributed to chemical degradation of the material [16,38,41]. These small variations are thought to arise from the physical interfacial interactions between the nanoparticles and the PCM chains, the possible role of the TiN nanoparticles as heterogeneous nucleation sites during solidification, and the partial restriction of PCM molecular mobility. When the melting and solidification enthalpies are examined, a non-monotonic dependence on the TiN content is observed. In the 1st cycle, while the ΔHm value of pristine RT70 HC was 280 J/g, the corresponding values for RT70 HC_0.1TiN and RT70 HC_0.5TiN increased to 298 and 306 J/g, respectively, and then decreased to 278, 276, and 267 J/g for RT70 HC_1.0TiN, RT70 HC_1.5TiN, and RT70 HC_2.0TiN, respectively. A similar trend was observed for the ΔHs values, which were 275 J/g for pristine RT70 HC and 282, 296, 264, 262, and 252 J/g for the 0.1, 0.5, 1.0, 1.5, and 2.0 wt.% TiN samples, respectively. Accordingly, the highest latent heat values were obtained not for pristine RT70 HC but for the RT70 HC_0.5TiN sample. This kind of non-monotonic variation, in which the latent heat first increases at low additive contents and then decreases at higher contents, has also been reported for nanoparticle-enriched paraffinic PCM systems and is closely related to the competition between nucleation-induced crystallization and the dilution of the active PCM phase [42]. The initial increase at low additive ratios may be attributed to a heterogeneous nucleation effect of the distributed TiN nanoparticles, which act as nucleation sites and promote a more ordered crystalline arrangement of the paraffinic chains, thereby increasing the effective crystallinity of the composite and the associated latent heat [43,44]. In the 1st cycle, the ωm values for RT70 HC_0.1TiN and RT70 HC_0.5TiN were therefore calculated as 106.43% and 109.29%, respectively, both exceeding 100% and indicating the increased latent heat storage capacity at low TiN contents. However, as the additive ratio increased beyond 0.5 wt.%, the latent heat values gradually fell below that of pristine RT70 HC. In the 1st cycle, the ωm values for RT70 HC_1.0TiN, RT70 HC_1.5TiN, and RT70 HC_2.0TiN were calculated as 99.29%, 98.57%, and 95.36%, respectively, while the corresponding ωs values were 96.00%, 95.27%, and 91.64%. This decrease at higher additive contents is governed by the mass replacement effect, a well-known physical phenomenon in nanocomposite PCM systems in which the addition of an increasing amount of the inactive inorganic TiN phase reduces the relative fraction of active paraffinic PCM available for latent heat storage [22,24,37]. Moreover, at higher loadings, the increased nanoparticle content and local clustering tendency may act as a physical barrier that restricts the mobility and flexibility of the paraffinic molecular chains during melting and crystallization. This behavior is also consistent with the SEM, EDX, and elemental mapping results. Overall, these results indicate the existence of an optimum low additive range in which a nucleation-related contribution could partly offset the mass replacement effect, with the 0.1–0.5 wt.% TiN range providing the highest latent heat storage capacity, while higher loadings progressively shift the balance toward enthalpy reduction [42,44]. In terms of thermal cycling stability, the melting and solidification peaks were preserved in all samples even after the 500th and 1000th cycles. For pristine RT70 HC, the ΔHm value decreased from 280 J/g in the 1st cycle to 267 J/g after the 500th cycle and to 259 J/g after the 1000th cycle. Similarly, the ΔHs value decreased from 275 J/g to 257 J/g after the 500th cycle and to 254 J/g after the 1000th cycle. This decrease can be associated with small changes in the crystallization order during long-term cycling, the effect of the repeated heating-cooling processes, and microstructural rearrangements that may occur during repeated phase transitions. Nevertheless, the fact that pristine RT70 HC retained relatively high enthalpy values even after 1000 cycles shows that the commercial PCM exhibits good cycling stability. An acceptable latent heat storage capacity was also preserved in the low-additive samples after 1000 cycles. The ΔHm values of RT70 HC_0.1TiN and RT70 HC_0.5TiN after 1000 cycles were determined to be 267 and 286 J/g, respectively, while the corresponding ΔHs values were 255 and 268 J/g. Notably, the RT70 HC_0.5TiN sample retained the highest latent heat values after 1000 cycles, with ωm and ωs values of 102.14% and 97.45%, respectively, both remaining at or above the initial level of pristine RT70 HC. This suggests that the enhanced latent heat in the low-additive samples was maintained during long-term cycling and that the addition of TiN at low concentrations did not disrupt the phase change behavior of RT70 HC [44]. In contrast, the post-cycling enthalpy loss became more pronounced at higher additive ratios. For the RT70 HC_1.0TiN sample, the ΔHm and ΔHs values after 1000 cycles decreased to 245 and 242 J/g, while for the RT70 HC_1.5TiN sample they decreased to 228 and 223 J/g. The most pronounced decrease was observed for the RT70 HC_2.0TiN sample, for which the ΔHm and ΔHs values after 1000 cycles dropped to 213 and 212 J/g, and the corresponding ωm and ωs values decreased to 76.07% and 77.09%, respectively. This decrease is thought to be associated with the reduction in the active PCM fraction, the increased nanoparticle-nanoparticle interactions, the possible local agglomeration regions, and the partial restriction of the crystallization mobility of the PCM molecules [40,45]. When the supercooling behavior is examined, the ΔT value of pristine RT70 HC was 7.2 °C in the 1st cycle. In the same cycle, the ΔT values of the TiN-added samples ranged from 6.8 to 8.5 °C. These relatively low and closely spaced ΔT values indicate that the TiN nanoparticles can partially act as nucleation sites during solidification, while the slightly higher ΔT value observed in some samples may be related to the increased local particle density and the partial restriction of PCM molecular mobility. After 1000 cycles, the ΔT values of all samples remained within a narrow range of approximately 6.6–7.6 °C, indicating that the supercooling behavior of the RT70 HC_TiN nanocomposite PCMs remained stable during long-term cycling. It should be noted, however, that the supercooling did not decrease systematically with TiN addition, since the 0.1 wt.% sample showed the largest first-cycle ΔT (8.5 °C), so the heterogeneous-nucleation interpretation is offered with caution and would benefit from crystallinity or crystallization-rate analysis for confirmation. Overall, the DSC results show that TiN addition largely preserved the phase change temperatures of RT70 HC, while affecting the melting and solidification enthalpies in a non-monotonic manner depending on the additive ratio and the number of cycles. At low additive ratios, especially in the 0.1–0.5 wt.% TiN range, the latent heat storage capacity of RT70 HC was even increased, consistent with a heterogeneous-nucleation contribution, and satisfactory cycling stability was achieved. At higher additive ratios, the enthalpy values decreased because of the dominance of the mass replacement effect and the reduction in the active PCM fraction. The high enthalpy retention and stable phase change temperatures obtained at low TiN contents indicate that these nanocomposites have strong potential for medium-temperature thermal energy storage (TES) and thermal management (TM) applications.

To separate the individual contribution of the SDS surfactant from that of the TiN nanoparticles, a surfactant-only control sample consisting of pristine RT70 HC with 4.0 wt.% SDS and no TiN (corresponding to the highest SDS level used in the composites) was additionally prepared under identical conditions and characterized by DSC. This control showed a melting peak temperature (Tpm) of 72.2 °C and a melting enthalpy (ΔHm) of 270.2 J/g, close to the 72.4 °C and 280 J/g of pristine RT70 HC, which corresponds to only a 3.5% reduction in ΔHm. Its supercooling degree (ΔT = 6.9 °C) was also close to that of pristine RT70 HC (7.2 °C). These small differences indicate that the SDS surfactant by itself does not markedly change the phase-change temperatures or the latent heat of RT70 HC, so the DSC behavior of the composites is governed mainly by the TiN nanoparticles.

### 2.6. Thermal Conductivity

**Figure 8:**
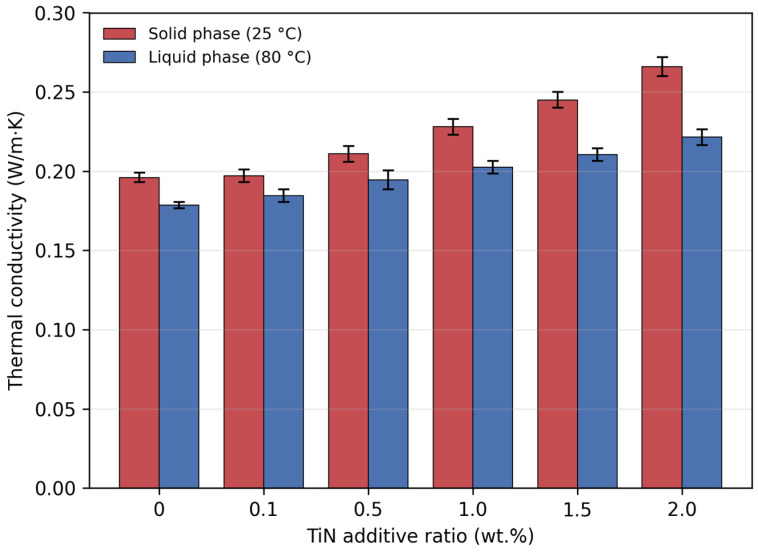
Thermal conductivity of the pristine RT70 HC and RT70 HC_TiN nanocomposite PCMs in the solid phase (25 °C) and the liquid phase (80 °C) as a function of the TiN additive ratio. The error bars represent the standard deviation of three measurements.

The thermal conductivity results of the RT70 HC and RT70 HC_TiN nanocomposite PCMs are presented in Figure 8. The KE% values calculated relative to pristine RT70 HC are reported below. Thermal conductivity is one of the most important thermophysical parameters determining the rate at which the stored heat is transferred in PCM-based systems. Although organic PCMs generally exhibit high latent heat storage capacities, their intrinsically low thermal conductivity can slow down the charging and discharging processes, and this remains one of the main bottlenecks limiting their practical use [7,22]. Therefore, in additive-enriched PCM systems, the aim is not only to preserve high enthalpy values but also to develop a composite structure that can accelerate heat transfer. In this study, the thermal conductivity was measured in both the solid phase (25 °C) and the liquid phase (80 °C), and each reported value represents the average of three measurements. The thermal conductivity of pristine RT70 HC was measured as 0.1785 W/(m·K). This value is consistent with the low-thermal-conductivity behavior commonly reported for organic/paraffin-based PCMs and is also close to the reported thermal conductivity value of RT70 HC of approximately 0.2 W/(m·K) [16,22].

With the addition of TiN, the thermal conductivity values of all nanocomposites increased markedly compared with pristine RT70 HC. The measured average values were 0.1845 W/(m·K) for RT70 HC_0.1TiN, 0.1945 W/(m·K) for RT70 HC_0.5TiN, 0.2025 W/(m·K) for RT70 HC_1.0TiN, 0.2105 W/(m·K) for RT70 HC_1.5TiN, and 0.2215 W/(m·K) for RT70 HC_2.0TiN. The corresponding solid-phase values, measured at 25 °C, were 0.196 W/(m·K) for pristine RT70 HC and 0.197, 0.211, 0.228, 0.245, and 0.266 W/(m·K) for the 0.1, 0.5, 1.0, 1.5, and 2.0 wt.% TiN samples, respectively. The solid-phase conductivity was consistently higher than the liquid-phase value for each composition, and its enhancement relative to pristine RT70 HC reached about 35.7% at 2.0 wt.% TiN, compared with about 24.1% in the liquid phase. Because the solid-phase conductivity governs the discharge (solidification) step during thermal-storage operation, this larger solid-phase enhancement is directly relevant to the target applications. Both data sets are compared in Figure 8. This gradual increase indicates that the TiN nanoparticles created additional heat conduction regions within the RT70 HC matrix and contributed to more effective heat transfer throughout the composite structure [16,40]. The high intrinsic thermal conductivity of TiN, together with its uniform distribution within the paraffinic matrix, is considered to be the main reason for this improvement [16,40]. The magnitude of the enhancement is broadly consistent with effective-medium models for particulate composites, in which the effective conductivity increases with the filler content but is limited by the interfacial thermal resistance between the nanoparticles and the matrix [46]. The KE% values also increased gradually with increasing additive ratio. The KE% values calculated relative to pristine RT70 HC were 3.36%, 8.96%, 13.45%, 17.93%, and 24.09% for the 0.1, 0.5, 1.0, 1.5, and 2.0 wt.% TiN samples, respectively. The highest KE% value of 24.09% was obtained for the 2.0 wt.% TiN sample. Especially at higher additive loadings, the increased probability of contact between the TiN nanoparticles may allow heat transfer to occur not only through the PCM phase but also through nanoparticle-assisted conduction networks, in which the distributed nanoparticles form more continuous thermal transport paths within the matrix [22,24,47,48]. The SEM images and elemental mapping results showed that the TiN nanoparticles were dispersed within the RT70 HC matrix and that the Ti and N signals became more intense with increasing additive ratio. Therefore, the gradual increase in thermal conductivity is thought to be related to the increasing amount of TiN within the matrix and the physical heat conduction contribution provided by the nanoparticles [38,39]. In this study, the thermal conductivity increased approximately linearly with increasing TiN content over the investigated 0.1–2.0 wt.% range (a linear fit gives k = 0.0201w + 0.182 W/(m·K), R^2^ = 0.98, where w is the TiN content in wt.%), without any sign of saturation. This behavior differs from the behavior frequently reported for nanocomposite PCM systems with higher loadings, in which the rate of increase tends to decrease at high additive contents because of interfacial thermal resistance, the local clustering tendency, and the boundary regions between the PCM and the nanoparticles [7,38]. The low error bars shown in Figure 8 support the reliability of the thermal conductivity measurements. The y-error values remained within a narrow range of approximately 0.0005–0.0105 W/(m·K), indicating good measurement stability among the samples. These error bars represent the standard deviation of the three repeated measurements on the same sample and therefore reflect measurement repeatability rather than reproducibility between independently prepared batches. When the present improvement is compared with the literature (Table 3), the moderate KE% values obtained with TiN are consistent with the general behavior of nanoparticle-enriched PCM systems, in which nitride- and metal-based additives can provide effective conduction contributions, but the overall improvement levels are strongly determined by the additive loading and the dispersion quality [40,49]. In this respect, the maximum KE% value of 24.09% obtained only at 2.0 wt.% TiN can be regarded as a meaningful improvement in overcoming the low-thermal-conductivity limitation of RT70 HC, especially considering that this improvement was achieved at low additive contents that largely preserve the latent heat storage capacity. While the thermal conductivity increases gradually with increasing additive ratio, the latent heat values decrease at higher additive loadings because of the reduction in the active RT70 HC fraction. Therefore, although the 2.0 wt.% TiN sample provided the highest thermal conductivity, the 0.1–0.5 wt.% range may offer a more balanced performance, with the 0.5 wt.% sample in particular combining a meaningful thermal conductivity improvement with the highest latent heat retention compared with pristine RT70 HC. These findings confirm that TiN addition improved the heat transfer performance of RT70 HC and that the RT70 HC_TiN nanocomposite PCMs prepared by the two-stage SDS-assisted method are suitable for medium-temperature TES and TM applications [16,49].

To confirm that the thermal-conductivity enhancement originates from the TiN nanoparticles rather than from the SDS surfactant, the same surfactant-only control (pristine RT70 HC with 4.0 wt.% SDS and no TiN) was measured under the same conditions. Relative to pristine RT70 HC, this control showed only a marginal thermal-conductivity change of about +3% in both the solid and liquid phases, which is far below the enhancement obtained with TiN (for example, about 35.7% in the solid phase at 2.0 wt.% TiN). This confirms that the surfactant contributes negligibly to heat conduction and that the measured conductivity improvement is attributable to the TiN nanoparticles. This behavior is also consistent with previous reports showing that surfactant addition does not increase, and may slightly reduce, the thermal conductivity of the base medium [50].

From a practical standpoint, TiN occupies an intermediate position among PCM nano-additives in terms of cost. It is more expensive than the common ceramic oxides such as Al_2_O_3_, TiO_2_, CuO, and SiO_2_, but considerably less expensive than carbon nanostructures such as graphene and carbon nanotubes [51]. Because the present formulations use only low loadings (up to 2 wt.%), the additive accounts for a small fraction of the total material cost, so the incremental cost per unit of stored energy remains limited. The use of TiN rather than a cheaper oxide is motivated by its combination of high intrinsic thermal conductivity, oxidation resistance, and ceramic-like thermal and chemical stability, together with its broadband optical absorption, a property set that the common oxide fillers do not offer simultaneously. It should also be noted that the density of TiN (about 5.2 g/cm^3^) is much higher than that of molten RT70 HC (about 0.77 g/cm^3^), which makes the nanoparticles prone to gravitational settling in the low-viscosity melt. The SDS surfactant and the two-stage ultrasonication were used to counteract this tendency, and no macroscopic sedimentation was observed during preparation or after cycling. Nevertheless, the long-term dispersion stability under repeated melting remains to be verified quantitatively, as noted in the limitations at the end of Section 2.8.

**Table 3:** Comparison of the thermal-conductivity enhancement of the present RT70 HC_TiN nanocomposite with related paraffin- and PCM-based systems reinforced with ceramic or hybrid nanofillers.

PCM Matrix	Nanofiller	Loading	Max. k (W/(m·K))	k Enhancement	Ref.
RT70 HC	TiN	2.0 wt.%	0.266 (solid)	+35.7%	This work
RT64 HC	HfC	2.0 wt.%	0.367 (solid)	+74.8%	[18]
RT54 HC	cBN	2.0 wt.%	0.312 (solid)	+59.2%	[19]
RT-35HC	TiO_2_	2.0 wt.%	0.341 (solid)	+59.5%	[13]
Paraffin	CeO_2_	2.0 wt.%	0.376 (solid)	+108.9%	[52]
Paraffin (PW70)	Graphene-Ag	0.3 wt.%	0.219 (solid)	+11%	[53]
Pentaerythritol (solid–solid)	TiN	0.2 wt.%	0.88 (solid)	+109.5%	[16]
Paraffin/TiO_2_ microcapsules	TiN/CNT	coating	0.513 (solid)	+151.5%	[12]

Values are taken from the cited studies. Where indicated, k refers to the solid phase, and the k enhancement is the increase in thermal conductivity relative to the corresponding pristine PCM reported in each source.

### 2.7. Specific Heat Capacity

**Figure 9:**
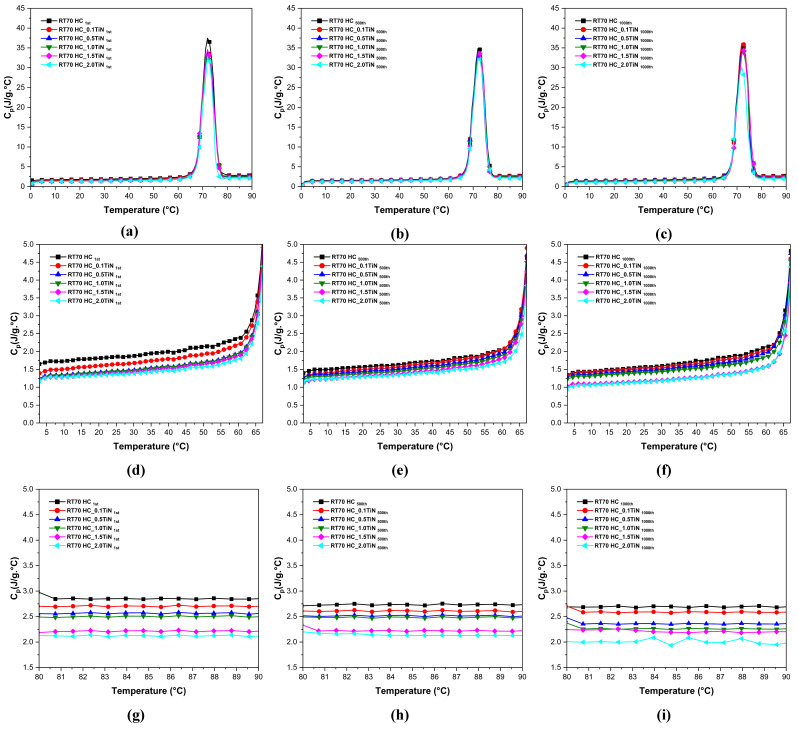
Temperature-dependent specific heat capacity (Cp) curves of the pristine RT70 HC and RT70 HC_TiN nanocomposite PCMs after the 1st, 500th, and 1000th cycles in (**a**–**c**) the full temperature range, (**d**–**f**) the solid-phase region, and (**g**–**i**) the liquid-phase region.

**Figure 10:**
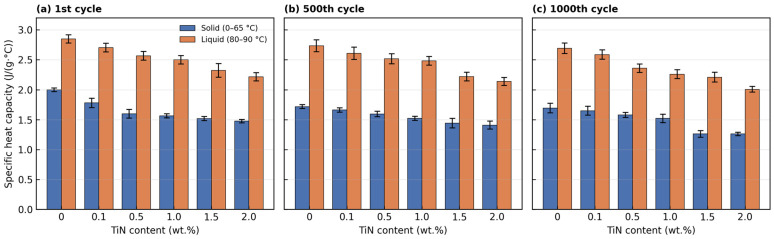
Average specific heat capacity (Cp) values of the pristine RT70 HC and RT70 HC_TiN nanocomposite PCMs in the solid and liquid phases after the (**a**) 1st, (**b**) 500th, and (**c**) 1000th cycles.

The temperature-dependent specific heat capacity behavior of the pristine RT70 HC and RT70 HC_TiN nanocomposite PCMs is presented in Figure 9. The average Cp values calculated for the solid- and liquid-phase regions after the 1st, 500th, and 1000th cycles are compared in Figure 10a, Figure 10b, and Figure 10c, respectively, and are listed in Table 4. To minimize the effect of the phase transition region, the average Cp values were calculated using the data in the 0–65 °C temperature range for the solid phase and the 80–90 °C temperature range for the liquid phase. The average Cp value of pristine RT70 HC in the 1st cycle was determined to be 2.00 J/(g·°C) in the solid phase and 2.85 J/(g·°C) in the liquid phase. These values are consistent with the typical thermal storage properties and Cp ranges reported for commercial organic RT-series paraffins [54]. With the addition of TiN, the measured Cp values were lower than those of pristine RT70 HC in both the solid and liquid phases. In the 1st cycle, the average solid-phase Cp values for the samples containing 0.1, 0.5, 1.0, 1.5, and 2.0 wt.% TiN were 1.78, 1.60, 1.56, 1.52, and 1.47 J/(g·°C), respectively. The average liquid-phase Cp values for the same additive ratios were 2.70, 2.57, 2.50, 2.32, and 2.22 J/(g·°C), respectively. In the measured data, the lowest Cp values in both phases were obtained for the 2.0 wt.% TiN sample. A reduction in the sensible heat capacity with inorganic-filler addition is expected in principle from the replacement of part of the high-heat-capacity organic paraffin by the low-heat-capacity TiN phase. For the small TiN fractions used here, however, this mass-weighted (rule-of-mixtures) contribution would amount to well below 1%, whereas the measured reductions are much larger, so they cannot be explained by mass replacement alone [16,54,55]. When the magnitude of this decrease is examined, the solid-phase Cp values in the 1st cycle decreased by approximately 11.0%, 20.1%, 21.9%, 24.2%, and 26.3% for the samples containing 0.1, 0.5, 1.0, 1.5, and 2.0 wt.% TiN, respectively. For the liquid phase, the corresponding decreases were approximately 5.1%, 10.0%, 12.3%, 18.5%, and 22.3%, in the same order. The tabulated values show a decreasing trend in both phases with increasing TiN content, although, as discussed below, the size of these differences is comparable to the measurement uncertainty and is therefore not interpreted as a quantitative composition dependence. Restriction of PCM molecular mobility near the nanoparticle surfaces has been proposed as an additional contribution to a reduced effective heat capacity in such composites, but the present single-run data do not allow this contribution to be separated or quantified [37,54,56]. Nevertheless, even at the highest TiN loading, the RT70 HC_TiN nanocomposites still retained Cp values within a practical range for sensible heat storage, ensuring that the sensible heat reduction did not completely degrade the overall thermal performance of the composite PCMs [16,37,40]. When the cycling behavior is evaluated, the Cp values of all samples exhibited a slight decreasing trend with increasing number of cycles but remained within a relatively stable range. The solid-phase Cp value of pristine RT70 HC was 2.00 J/(g·°C) in the 1st cycle, 1.72 J/(g·°C) after the 500th cycle, and 1.69 J/(g·°C) after the 1000th cycle. In the liquid phase, the corresponding values were 2.85, 2.73, and 2.69 J/(g·°C), respectively. For an unmodified paraffin, an apparent reduction in this magnitude (about 14% in the solid phase over the first 500 cycles) has no clear physical origin, which indicates that the single-run ASTM E1269 determinations carry an uncertainty comparable to the differences measured between compositions and between cycles [5,54]. A similar decreasing trend with increasing number of cycles was also observed for the TiN-added samples. For example, for the 1.0 wt.% TiN sample, the solid-phase Cp value was 1.56 J/(g·°C) in the 1st cycle, 1.52 J/(g·°C) after the 500th cycle, and 1.52 J/(g·°C) after the 1000th cycle, while the corresponding liquid-phase Cp values were 2.50, 2.48, and 2.26 J/(g·°C), respectively. After the 500th cycle, the solid-phase Cp values of the TiN-added samples ranged from 1.41 to 1.66 J/(g·°C), while the liquid-phase Cp values ranged from 2.14 to 2.61 J/(g·°C). After the 1000th cycle, the solid-phase and liquid-phase Cp values were in the ranges of 1.26–1.65 J/(g·°C) and 2.01–2.59 J/(g·°C), respectively. These findings show that, although the Cp values gradually decreased during long-term cycling, the RT70 HC_TiN nanocomposites maintained a relatively stable sensible heat storage behavior and that the interfacial structure and the additive phase in these composites were not severely degraded during repeated thermal cycling [40,54]. When the Cp values in the solid and liquid phases are compared, the liquid-phase Cp values are higher than the solid-phase values for all samples. This behavior can be associated with the increased molecular mobility in the liquid phase and the ability of the system to absorb more energy in response to temperature changes. In terms of error bars, the y-error values shown in Figure 10 are generally low. In the solid phase, the error values mostly remained in the range of approximately 0.028–0.081, while in the liquid phase they were generally between approximately 0.047 and 0.115. The relatively higher error values observed in some samples may be related to the local heterogeneity or particle-rich regions arising from the increased TiN content. These error bars reflect the repeatability of repeated readings on the same subsample rather than reproducibility between independently prepared batches. Taken together with the rule-of-mixtures and internal-control considerations noted above, the absolute Cp values and the percentage differences reported here and in Table 4 are provided for completeness but are treated qualitatively, and the quantitative composition- and cycle-dependent Cp comparison is not used to draw mechanistic conclusions (see the limitations at the end of Section 2.8). When evaluated together with the DSC and thermal conductivity results, TiN addition produces a multifunctional but trade-off-based thermophysical effect on RT70 HC. While TiN increased the thermal conductivity at all additive ratios, it reduced both the latent heat and the sensible heat capacity because of the replacement of the active organic PCM fraction by the inorganic TiN phase. Therefore, the Cp results indicate that the optimum additive ratio in the RT70 HC_TiN nanocomposites should be determined by considering not only the thermal conductivity improvement but also the accompanying reductions in the sensible and latent heat capacities together with the cycling stability. Nevertheless, the relatively stable Cp behavior retained by the nanocomposite samples after 1000 cycles shows that the prepared nanocomposite PCMs maintained a reliable, albeit reduced, sensible heat storage capability for long-term TES and TM applications.

For the same surfactant-only control (pristine RT70 HC with 4.0 wt.% SDS and no TiN), the average Cp values were 1.85 J/(g·°C) in the solid phase and 2.67 J/(g·°C) in the liquid phase, corresponding to reductions of about 6.6% and 6.4%, respectively, relative to pristine RT70 HC measured under the same conditions. These reductions are much smaller than those measured for the TiN-containing composites (up to about 26% in the solid phase), but they are not negligible, which indicates that part of the apparent Cp decrease in the composites is associated with the SDS surfactant and the dilution of the paraffin rather than with the TiN phase alone. This observation is consistent with the qualitative treatment of the Cp data adopted here.

### 2.8. Thermal Stability and Reliability

**Figure 11:**
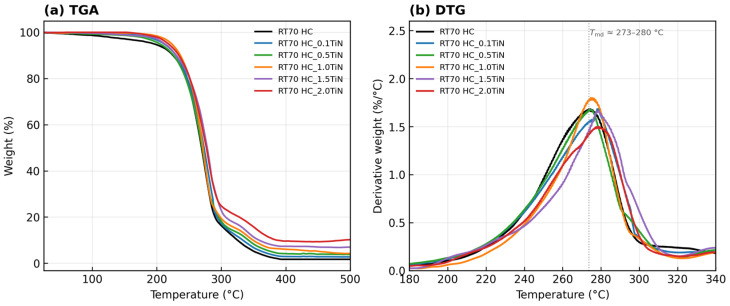
(**a**) TGA and (**b**) DTG curves of the pristine RT70 HC and RT70 HC_TiN nanocomposite PCMs, measured under nitrogen up to 500 °C.

The thermal degradation behavior of the pristine RT70 HC and RT70 HC_TiN nanocomposite PCMs was examined by TGA, and the obtained TGA and DTG curves are presented in Figure 11. The degradation onset temperature (Tod), maximum degradation temperature (Tmd), and residual weight percentage (Rw) values obtained from these curves are listed in Table 5. As shown in Figure 11, the pristine RT70 HC and the TiN-added nanocomposite PCMs did not exhibit any significant mass loss up to approximately 200 °C. This indicates that RT70 HC remained thermally stable up to a temperature range well above its working phase change temperature of approximately 72 °C. The Tod and Tmd values of pristine RT70 HC were determined to be 240.4 °C and 273.7 °C, respectively. These values are consistent with the thermal degradation behavior reported in the literature for paraffin-based PCMs [22]. For the TiN-added samples, the Tod values were generally close to or higher than that of pristine RT70 HC. The Tod values of RT70 HC_0.1TiN, RT70 HC_0.5TiN, RT70 HC_1.0TiN, RT70 HC_1.5TiN, and RT70 HC_2.0TiN were determined to be 244.6, 240.8, 247.1, 248.0, and 244.3 °C, respectively. The highest Tod value of 248.0 °C was obtained for the RT70 HC_1.5TiN sample. This increase can be explained by the role of the TiN nanoparticles as a thermally stable inorganic phase that provides thermal resistance within the RT70 HC matrix. When the Tmd values are examined, the maximum degradation temperature of pristine RT70 HC of 273.7 °C changed only slightly after TiN addition. The Tmd values of RT70 HC_0.1TiN, RT70 HC_1.5TiN, and RT70 HC_2.0TiN increased to 279.8, 280.0, and 279.6 °C, respectively, while the values of RT70 HC_0.5TiN and RT70 HC_1.0TiN remained close to that of pristine RT70 HC at 273.4 and 272.5 °C, respectively. The general trend toward higher Tmd values at several additive ratios indicates that the nanocomposite structure became somewhat more resistant to thermal degradation after TiN addition. This behavior is consistent with the nanoparticle dispersion observed in the SEM, EDX, and elemental mapping analyses [38,40]. The slight non-monotonic variation in the Tmd values may be related to the local distribution of the nanoparticles within the matrix and the formation of different degradation regions at the PCM-nanoparticle interfaces. For certain nanoparticle distributions, some PCM molecules may be confined near the nanoparticle surfaces. This can cause molecular groups with different kinetic energies to degrade over slightly different temperature ranges [16]. Therefore, the small fluctuations in Tmd do not necessarily indicate a decrease in thermal stability. Rather, they show that the degradation pathway changed slightly depending on the TiN content and its local distribution. The Rw values most directly reveal the effect of TiN addition. While the Rw value of pristine RT70 HC was only 1.68%, the TiN-added samples exhibited a systematic increase in Rw with increasing additive ratio. The Rw values of RT70 HC_0.1TiN, RT70 HC_0.5TiN, RT70 HC_1.0TiN, RT70 HC_1.5TiN, and RT70 HC_2.0TiN were obtained as 2.91%, 4.14%, 6.04%, 7.33%, and 9.60%, respectively. This monotonic increase is mainly attributed to the thermally stable inorganic nature of TiN, which remains as a residual phase after the degradation of the organic PCM at high temperatures. It should be noted, however, that the residual masses exceed the nominal TiN contents (for example, 9.60% at 400 °C for the 2.0 wt.% TiN sample versus a nominal 2.0 wt.%). Because the residue does not decrease further between 400 and 500 °C (Figure 11), this excess does not arise from incomplete volatilization but is instead attributed to the paraffin char (about 1.68% for the pristine PCM), the inorganic ash from the SDS surfactant, and the likely local enrichment of the dense TiN particles in the small (about 10 mg) subsample. The residual mass is therefore treated as a qualitative rather than a strictly quantitative measure of the TiN content. The clear and systematic correlation between the residual weight and the nominal TiN content is consistent with the incorporation of the intended TiN amounts into the composite structure [16,40]. Although the DSC and Cp results show a decrease in the latent and sensible heat values at higher TiN contents, the TGA results show that TiN can improve the degradation resistance and increase the residual mass. Taken together, the degradation onset temperatures of the RT70 HC_TiN nanocomposite PCMs, which remained above approximately 240 °C, indicate that these materials possess a wide thermal safety margin compared with the RT70 HC phase change temperature of approximately 72 °C. This temperature range provides sufficient thermal stability for TES and TM applications operating at medium temperature levels [40,57].

**Figure 12:**
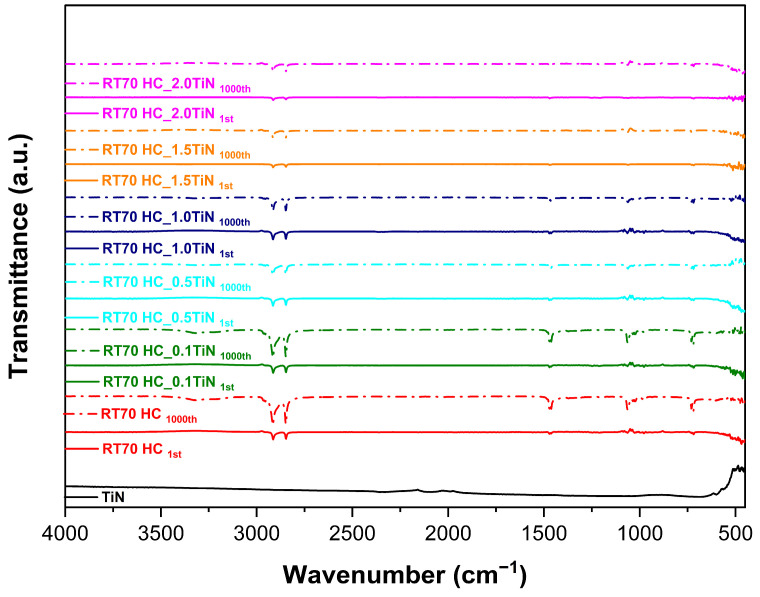
FT-IR spectra of the TiN, pristine RT70 HC, and RT70 HC_TiN nanocomposite PCMs before and after thermal cycling.

The FT-IR spectra of the pristine RT70 HC and RT70 HC_TiN nanocomposite PCMs before and after 1000 thermal cycles are presented in Figure 12. As shown in Figure 12, the characteristic absorption bands of the pristine RT70 HC and RT70 HC_TiN nanocomposite PCMs after 1000 cycles remained almost identical to those observed before thermal cycling. The characteristic RT70 HC bands observed at approximately 2914, 2846, 1471, and 719 cm^−1^ were preserved after long-term cycling, with the band positions changing by at most 1 cm^−1^ (Table 6) in both the pristine PCM and the TiN-added nanocomposites. No new absorption peaks or loss of existing peaks were observed after cycling. This result indicates that no significant chemical degradation or new chemical bond formation occurred during the repeated thermal processes and confirms the chemical stability of the developed nanocomposite PCM system [40,58]. Although very slight peak shifts were observed after thermal cycling, these shifts remained negligible, and the overall spectral profile was preserved. Moreover, the characteristic RT70 HC bands remained visible in all TiN-added samples even after 1000 cycles. These FT-IR findings are consistent with the DSC and TGA results. While the DSC analysis showed that the phase change temperatures of the nanocomposite PCMs remained relatively stable even after 1000 cycles, the TGA analysis confirmed that the thermal degradation behavior of the TiN-added samples remained within a stable temperature range well above the working temperature. Together with the FT-IR results, these findings show that the developed RT70 HC_TiN nanocomposite PCMs preserved not only their thermophysical properties but also their chemical structures after repeated thermal cycling. Moreover, the preservation of the characteristic RT70 HC absorption bands after cycling further supports the previous FT-IR, XRD, SEM, and EDX analyses, which showed that RT70 HC and the TiN nanoparticles combined physically without forming new chemical phases. Therefore, the thermal cycling FT-IR results show that the two-stage SDS-assisted method enabled the formation of chemically stable RT70 HC_TiN nanocomposite PCMs that can maintain their structural integrity during long-term thermal processing. Taken together, the FT-IR spectra after 1000 cycles confirm that the developed RT70 HC_TiN nanocomposite PCMs possess good chemical reliability and long-term thermal cycling stability. These results show that the prepared nanocomposites are suitable for long-term TES and TM applications operating under repeated thermal cycling conditions.

The present study has several limitations that also indicate directions for future work. First, a single batch was prepared for each composition, so the reported values reflect measurement repeatability rather than batch-to-batch reproducibility, and independent replicate batches for the key compositions would be required to establish the statistical confidence of the optimum range. Second, a surfactant-only control (RT70 HC with 4.0 wt.% SDS but without TiN) was prepared and characterized, and it showed only minor changes relative to pristine RT70 HC (a thermal-conductivity change of about +3%, a 3.5% reduction in melting enthalpy, and Cp reductions of about 6–7%), which confirms that the property changes reported here originate mainly from the TiN nanoparticles rather than from the SDS surfactant, although a full composition-resolved deconvolution of the surfactant contribution across all TiN loadings was not attempted. Third, the thermal conductivity was measured at two discrete temperatures (25 °C in the solid phase and 80 °C in the liquid phase) rather than continuously through the melting range, so the conductivity behaviour within the phase-transition region was not resolved. Fourth, the specific heat capacity was obtained from single ASTM E1269 runs without a reported uncertainty, so the Cp comparisons between compositions should be regarded as indicative, and triplicate measurements with calibration are required for a quantitative comparison. Fifth, the TGA was performed under nitrogen up to 500 °C, so the thermal-stability conclusions apply to an inert atmosphere, and the oxidative (air) stability and the residual behaviour above 500 °C remain to be assessed. Sixth, because of the large density difference between TiN (about 5.2 g/cm^3^) and molten RT70 HC (about 0.77 g/cm^3^), long-term sedimentation or phase separation under repeated melting cannot be excluded, and although no macroscopic separation was observed, a direct dispersion-stability test was not carried out. Finally, the completeness of every melting-solidification transition in the 0.5 g charge during accelerated cycling was not independently verified with an embedded thermocouple, and transient melting/solidification and photothermal (solar) characterization are left for future work. Accordingly, the post-cycling reduction in latent heat is interpreted as an overall indicator of the total thermal effect of accelerated aging and of long-term stability, rather than as evidence that every one of the 1000 cycles reached complete melting, and strict full melting in each cycle is not a requirement of the present accelerated-aging protocol.

## 3. Materials and Methods

### 3.1. Materials

In this study, commercial RT70 HC was used as the base PCM, while TiN nanoparticles were used as the nano-additive phase. The main supplier information and selected material properties are summarized in Table 7.

### 3.2. Preparation of the RT70 HC_TiN Nanocomposite PCMs

**Figure 13:**
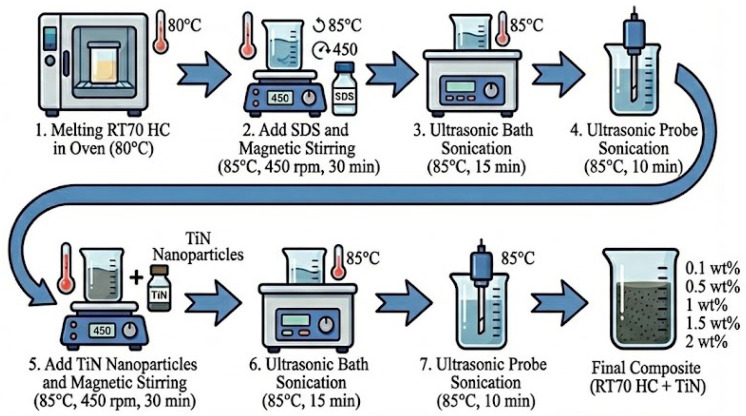
Schematic representation of the preparation process of the RT70 HC_TiN nanocomposite PCMs using melting, magnetic stirring, bath ultrasonication, and probe ultrasonication steps.

The preparation route of the RT70 HC_TiN nanocomposite PCMs is shown schematically in Figure 13. In this study, five TiN-containing nanocomposite formulations were produced using pristine RT70 HC and nominal additive ratios ranging between 0.1 and 2 wt.%. The sample codes, RT70 HC_TiN ratios, and composition information are summarized in Table 8. A single batch was produced for each composition, without preparing replicate samples. The calculated amount of RT70 HC was first placed in a glass beaker and melted in a laboratory oven preheated to 80 °C, well above the melting temperature range of RT70 HC. After complete melting, sodium dodecyl sulfate (SDS) was added to the molten RT70 HC at a 2:1 mass ratio relative to the TiN nanoparticles in order to improve the wettability of the nanoparticle phase and to minimize possible agglomeration during dispersion [50]. The nominal TiN contents given in Table 8 are defined relative to the RT70 HC + TiN basis, and the SDS added at this 2:1 SDS:TiN ratio (0–4 wt.% relative to that basis) is reported separately in Table 8 rather than being included in the 100 wt.% normalization. The SDS-containing RT70 HC mixture was then magnetically stirred at 450 rpm and 85 °C for 30 min, followed by bath ultrasonication at 85 °C for 15 min and probe ultrasonication at 85 °C for 10 min at 20% amplitude. In the second stage, the calculated amount of TiN nanoparticles was added to the SDS-pretreated RT70 HC, and the same sequence of magnetic stirring (85 °C, 450 rpm, 30 min), bath ultrasonication (85 °C, 15 min) and probe ultrasonication (85 °C, 10 min, 20% amplitude) was applied to ensure a homogeneous dispersion of the TiN nanoparticles within the RT70 HC matrix [5]. This two-stage procedure was used to ensure the homogeneous dispersion of the TiN phase, to reduce the interfacial energy through the surface activity of SDS, and to minimize sedimentation during preparation. After the final probe ultrasonication step, the prepared RT70 HC_TiN mixtures were cooled to room temperature and solidified. The resulting nanocomposite PCMs were stored for the subsequent structural, morphological, and thermophysical characterization.

### 3.3. Structural and Morphological Characterization

X-ray diffraction (XRD) analysis was carried out to determine the crystal structure and phase composition of the pristine RT70 HC, TiN nanoparticles, and RT70 HC_TiN nanocomposite PCMs. The XRD patterns were recorded using a MiniFlex 600 diffractometer with CuKα radiation (λ = 1.54 Å) (Rigaku Corporation, Tokyo, Japan). The measurements were carried out over a 2θ range of 5–90° with a step width of 0.02° and a scan rate of 2.0°/min. The chemical structure, functional groups, and possible chemical interactions between RT70 HC and TiN were investigated by Fourier-transform infrared spectroscopy (FT-IR). The FT-IR spectra were acquired using a QATR-S FT-IR spectrometer (Shimadzu Corporation, Kyoto, Japan) over the wavenumber range of 400–4000 cm^−1^. Surface morphology observations were performed using a GeminiSEM 500 field-emission scanning electron microscope (Carl Zeiss, Oberkochen, Germany) operated at an accelerating voltage of 3.0 kV with an InLens detector. Because the organic PCM-based samples are electrically non-conductive, they were sputter-coated with a thin gold layer prior to SEM and EDX analysis to reduce charging effects. Accordingly, a low-intensity Au Mα peak at about 2.12 keV originating from this conductive coating appears in the EDX spectra. Because this peak arises from the coating rather than from the sample material, it is observed in all samples, including the SDS-free pristine RT70 HC and TiN references. Elemental composition analyses and elemental mapping were performed at an accelerating voltage of 20 kV using an EDAX TEAM energy-dispersive X-ray spectroscopy (EDX) system integrated into the GeminiSEM 500 instrument, in order to evaluate the spatial distribution of the C, N, O, and Ti elements within the RT70 HC_TiN nanocomposite structure. For each sample, the elemental composition reported in Table 1 was obtained from a representative area of the analyzed field at the imaging magnification, so the values are semi-quantitative and representative rather than statistically averaged over multiple regions.

### 3.4. Thermophysical Characterization and Thermal Cycling Tests

Prior to thermophysical characterization, the solidified RT70 HC and RT70 HC_TiN nanocomposite PCM samples were gently ground using an agate mortar to obtain a more homogeneous powder form, from which representative specimens of approximately 10 mg were taken for the DSC, Cp, and TGA measurements. For the post-cycling analyses, the entire cycled charge in each sealed tube was re-melted and re-ground before subsampling, so that the measured specimens represented the whole sample rather than a single layer. The melting and solidification behavior of the pristine RT70 HC and RT70 HC_TiN nanocomposite PCMs was investigated using a DSC7010 differential scanning calorimeter (Hitachi, Tokyo, Japan). Approximately 10 mg of each sample was placed in aluminum pans, and an empty aluminum pan was used as the reference. The DSC measurements were performed under a nitrogen atmosphere at a flow rate of 20 mL/min. The samples were heated and cooled between 0 and 80 °C at a rate of 5 °C/min. The DSC analyses were performed for the 1st, 500th, and 1000th thermal cycles to determine the onset, peak, and end temperatures of melting and solidification and the corresponding melting and solidification enthalpies. Specific heat capacity (Cp) measurements were also performed using the same DSC instrument over the temperature range of 0–90 °C at a heating rate of 5 °C/min under a nitrogen flow of 20 mL/min. Cp measurements were carried out by the three-run method according to ASTM E1269. In this procedure, three separate DSC measurements were performed: an empty aluminum pan baseline run, a sapphire standard run, and a sample run. The Cp values were calculated using the MUSE Standard Analysis software (version 9.21U; Hitachi High-Tech Science Corporation, Tokyo, Japan) based on the heat flow responses of the sample, the sapphire reference, and the empty pan. The average Cp values were evaluated separately for the solid and liquid regions. In this study, the 0–65 °C temperature range was considered as the solid-phase region, and the 80–90 °C temperature range as the liquid-phase region. The Cp analyses were performed for the 1st, 500th, and 1000th thermal cycles. Thermal stability was evaluated by thermogravimetric analysis using an MA500 TGA/DTA analyzer (Shimadzu Corporation, Kyoto, Japan). Approximately 10 mg of each sample was placed in platinum pans. The measurements were performed under a nitrogen atmosphere between 25 and 500 °C at a heating rate of 5 °C/min and a nitrogen flow rate of 40 mL/min. The TGA analyses were performed for the initial samples and repeated after 1000 thermal cycles in order to examine the effect of long-term cycling on the thermal degradation behavior. Accelerated thermal cycling tests were carried out using a SimpliAmp thermal cycler (Applied Biosystems, Thermo Fisher Scientific, Waltham, MA, USA). The instrument was programmed to cycle between 35 and 85 °C with a hold of about 5 s at each set-point temperature. Heating and cooling between the two set-points were controlled automatically by the instrument (maximum block ramp rate 4 °C/s); the actual rate experienced by the 0.5 g solid sample was slower and volume-limited, so each full melting-solidification cycle took appreciably longer than the block transition time. Approximately 0.5 g of each sample was placed in sealed tubes. The samples were subjected to repeated melting and solidification cycles between 35 and 85 °C for up to 500 and 1000 cycles. After the selected numbers of cycles, the samples were recharacterized by DSC and Cp measurements under the conditions described above. In addition, FT-IR analyses were repeated after 1000 cycles to evaluate possible changes in the chemical structure and thermal stability after long-term cycling. Thermal conductivity was measured in both the solid phase, at 25 °C on the solidified samples, and the liquid phase, at 80 °C on the fully molten samples. The solid-phase measurements used the KD2 Pro TR-1 single-needle sensor, which is intended for solid and granular materials, whereas the liquid-phase measurements used the KS-1 single-needle sensor, which is intended for liquids. The 80 °C liquid measurements were performed above the peak melting point to ensure reliable probe-sample contact under fully molten conditions. The molten samples were placed in 100 mL glass beakers to ensure sufficient contact between the probe and the liquid phase. For each composition and each phase, three thermal conductivity measurements were performed and averaged using a KD2 Pro thermal property analyzer (Decagon Devices, Inc., Pullman, WA, USA), which is based on the transient line heat source (hot-wire) principle [59]. The liquid-phase (KS-1) measurements were run in the sensor’s default low-power mode, which applies only a small heating power to the needle specifically to suppress free convection in the molten sample, whereas the solid-phase (TR-1) measurements used the sensor’s default high-power mode. The analyzer was calibrated on a regular basis following the manufacturer’s recommended verification procedure. A waiting period of 20 min was applied between successive measurements to allow both the sample and the probe to return to stable thermal conditions. The average value of the three measurements was reported as the final thermal conductivity for each composition. The normalized melting fraction (ωm) and the normalized solidification fraction (ωs) were calculated using the melting enthalpy (ΔHm) and solidification enthalpy (ΔHs) values, respectively. These parameters were used to determine the relative latent heat storage retention of the RT70 HC_TiN nanocomposite PCMs compared with pristine RT70 HC during the melting and solidification processes. The ωm and ωs values were calculated according to Equations (1) and (2), respectively [5,13]:

(1) $$\omega m=\frac{\Delta Hm(RT70\;HC\_TiN)}{\Delta Hm(RT70\;HC)}\times \;100$$

(2) $$\omega s=\frac{\Delta Hs(RT70\;HC\_TiN)}{\Delta Hs\;(RT70\;HC)}\times 100$$

where ΔHm (RT70 HC_TiN) and ΔHs (RT70 HC_TiN) denote the melting and solidification enthalpy values of the TiN-added RT70 HC nanocomposite PCMs, respectively, and ΔHm (RT70 HC) and ΔHs (RT70 HC) denote the corresponding melting and solidification enthalpy values of pristine RT70 HC. The degree of supercooling (ΔT) was calculated as the temperature difference between the peak melting temperature (Tpm) and the peak solidification temperature (Tps), as given in Equation (3) [5,13]:

ΔT = Tpm − Tps(3)

where Tpm and Tps represent the peak melting and peak solidification temperatures, respectively. The peak temperatures were used because the melting and crystallization peaks are the most consistently identifiable features of the DSC curves at the 5 °C/min heating rate, and the onset-based difference (Tbm − Tbs) follows the same qualitative trend across the sample set. To evaluate the effect of TiN addition on the heat-transfer-related thermophysical properties of RT70 HC, the thermal conductivity enhancement (KE%) and the specific heat capacity enhancement (CE%) were calculated relative to pristine RT70 HC. KE% was used to quantify the percentage increase in thermal conductivity, and CE% to determine the percentage change in specific heat capacity. These values were calculated according to Equation (4) [5,13]:

(4) $$CE\%/KE\%=\left(\frac{{(C}_{p}/k)RT70\;HC\_TiN\;-{(C}_{p}/k)RT70\;HC}{{(C}_{p}/k)RT70\;HC}\right)\times \;100$$

where k(RT70 HC_TiN) and k(RT70 HC) represent the thermal conductivity values of the TiN-added RT70 HC nanocomposite PCM and pristine RT70 HC, respectively, and Cp(RT70 HC_TiN) and Cp(RT70 HC) represent the specific heat capacity values of the TiN-added RT70 HC nanocomposite PCM and pristine RT70 HC, respectively. Positive KE% and CE% values indicate an improvement relative to pristine RT70 HC, whereas lower values indicate a weaker enhancement effect.

## 4. Conclusions

In this study, titanium nitride-reinforced RT70 HC_TiN nanocomposite PCMs were developed by adding 0.1–2.0 wt.% TiN nanoparticles to commercial RT70 HC using a two-stage method assisted by SDS, magnetic stirring, bath ultrasonication, and probe ultrasonication. The study aimed to tailor the structure–property relationship of a medium-temperature organic PCM using a thermally stable nitride nanophase for energy storage and thermal management under repeated thermal stress. FT-IR and XRD analyses confirmed that TiN was physically integrated into the RT70 HC matrix without the formation of new chemical bonds or secondary crystalline phases. SEM-EDX and elemental mapping indicated the concentration-dependent distribution of TiN within the PCM matrix, with the Ti content increasing systematically with increasing additive ratio. The DSC results showed that the phase change temperature range of RT70 HC was largely preserved after TiN addition and that the peak melting temperature remained around 72 °C at all additive ratios. The latent heat varied non-monotonically with TiN content: it increased at low loadings (0.1–0.5 wt.%), reaching the highest values for the 0.5 wt.% sample, and then decreased at higher loadings as the mass replacement effect became dominant. The 2.0 wt.% TiN sample retained ΔHm/ΔHs values of 267/252 J/g in the first cycle and 213/212 J/g after 1000 cycles, which still represent practically useful energy storage densities. The thermal conductivity increased gradually at all additive ratios, rising from 0.1785 W/(m·K) for pristine RT70 HC to 0.2215 W/(m·K) at 2.0 wt.% TiN in the liquid phase, corresponding to a maximum improvement of 24.09%, while the solid-phase conductivity showed an even larger improvement of about 35.7% at the same loading, which is directly relevant to the discharge step. In contrast, the measured specific heat capacity was lower at higher TiN loadings. Because these differences exceed the sub-1% mass-replacement prediction and lie within the measurement uncertainty, the Cp trend is reported qualitatively rather than as a quantitative composition-dependent effect. Nevertheless, the nanocomposites maintained a reliable, albeit reduced, sensible heat storage capability even after 1000 cycles. The TGA results, obtained under an inert nitrogen atmosphere, indicated degradation onset temperatures above approximately 240 °C, pointing to a wide thermal safety margin relative to the phase change range of approximately 72 °C, while the residual mass increased systematically with the TiN content, confirming the incorporation of the inorganic phase. The FT-IR spectra recorded after 1000 thermal cycles confirmed the preservation of the characteristic functional groups of RT70 HC, demonstrating the good chemical reliability and long-term cycling stability of the developed nanocomposite PCMs. Overall, TiN addition produced a multifunctional but trade-off-based thermophysical effect on RT70 HC, simultaneously enhancing the thermal conductivity and thermal stability while reducing the latent and sensible heat capacities. Therefore, the low additive range, especially 0.1–0.5 wt.% TiN, provided the most balanced performance in terms of latent heat retention, thermal conductivity improvement, sensible heat capacity, and cycling reliability. Because each composition was prepared as a single batch and characterized using small (about 10 mg) specimens, this optimum range and the associated low-loading latent-heat increase (up to about 9% at 0.5 wt.%) should be regarded as indicative rather than statistically proven, since they may fall within the subsampling variance, and independent replicate batches are required to establish statistical confidence. These results indicate that the RT70 HC_TiN nanocomposite PCMs show potential for medium-temperature TES, passive thermal regulation, solar-assisted heat storage, domestic hot-water systems, waste-heat recovery, and PV/T applications, where repeated phase transitions and long-term structural stability are required.

## Figures and Tables

**Table 1:** EDX results of TiN, RT70 HC, and the TiN-added RT70 HC nanocomposites.

Sample	C	N	O	Ti
TiN	-	23.81	-	76.19
RT70 HC	92.77	-	7.23	-
RT70 HC_0.1TiN	87.93	2.36	9.59	0.12
RT70 HC_0.5TiN	89.32	0.89	9.36	0.43
RT70 HC_1.0TiN	84.75	4.60	9.90	0.76
RT70 HC_1.5TiN	85.09	4.59	8.66	1.66
RT70 HC_2.0TiN	89.34	2.21	6.75	1.69

A dash (-) indicates that the element was not detected.

**Table 2:** DSC thermal parameters of the pristine RT70 HC and RT70 HC_TiN nanocomposite PCMs obtained after the 1st, 500th, and 1000th thermal cycles. The degree of supercooling (ΔT) was calculated from the unrounded peak temperatures according to Equation (3) and then rounded, so it may differ by up to 0.1 °C from the difference in the rounded Tpm and Tps values listed.

Sample	Tbm (°C)	Tpm (°C)	Tfm (°C)	ΔHm (J/g)	Tbs (°C)	Tps (°C)	Tfs (°C)	ΔHs (J/g)	ωm (%)	ωs (%)	ΔT (°C)
RT70 HC_1st_	68.7	72.4	75.3	280	67.4	65.2	61.9	275	100.00	100.00	7.2
RT70 HC_500th_	67.8	72.0	75.8	267	67.5	65.5	62.0	257	95.36	93.45	6.5
RT70 HC_1000th_	68.1	72.5	76.0	259	67.4	65.0	62.4	254	92.50	92.36	7.6
RT70 HC_0.1TiN_1st_	68.6	73.0	75.9	298	67.2	64.5	61.4	282	106.43	102.55	8.5
RT70 HC_0.1TiN_500th_	67.7	72.1	75.9	272	67.4	65.1	62.5	258	97.14	93.82	7.0
RT70 HC_0.1TiN_1000th_	67.9	72.3	75.9	267	67.4	65.1	62.0	255	95.36	92.73	7.2
RT70 HC_0.5TiN_1st_	67.8	72.1	76.0	306	67.4	65.2	62.5	296	109.29	107.64	6.8
RT70 HC_0.5TiN_500th_	67.7	72.2	76.0	289	67.4	65.1	62.0	265	103.21	96.36	7.1
RT70 HC_0.5TiN_1000th_	67.9	72.3	76.1	286	67.3	65.1	62.2	268	102.14	97.45	7.2
RT70 HC_1.0TiN_1st_	67.9	72.2	75.6	278	67.5	65.2	62.0	264	99.29	96.00	7.0
RT70 HC_1.0TiN_500th_	68.3	72.6	76.3	262	67.3	64.9	61.9	256	93.57	93.09	7.6
RT70 HC_1.0TiN_1000th_	68.3	72.1	74.7	245	67.5	65.3	62.5	242	87.50	88.00	6.7
RT70 HC_1.5TiN_1st_	67.8	72.3	76.1	276	67.4	65.0	61.7	262	98.57	95.27	7.4
RT70 HC_1.5TiN_500th_	68.5	72.1	74.8	250	67.6	65.4	62.5	246	89.29	89.45	6.7
RT70 HC_1.5TiN_1000th_	68.6	72.4	74.9	228	67.8	65.2	63.1	223	81.43	81.09	7.2
RT70 HC_2.0TiN_1st_	67.8	72.1	75.8	267	67.5	65.3	62.4	252	95.36	91.64	6.8
RT70 HC_2.0TiN_500th_	69.3	72.2	74.8	234	67.8	65.4	63.0	228	83.57	82.91	6.8
RT70 HC_2.0TiN_1000th_	67.8	71.9	75.3	213	67.4	65.3	62.3	212	76.07	77.09	6.6

**Table 4:** Average specific heat capacities (Cp) in the solid (0–65 °C) and liquid (80–90 °C) phases of the pristine RT70 HC and RT70 HC_TiN nanocomposite PCMs after the 1st, 500th, and 1000th thermal cycles.

Sample	Cp, Solid (J/(g·°C))	Cp, Liquid (J/(g·°C))
RT70 HC_1st_	2.00	2.85
RT70 HC_500th_	1.72	2.73
RT70 HC_1000th_	1.69	2.69
RT70 HC_0.1TiN_1st_	1.78	2.70
RT70 HC_0.1TiN_500th_	1.66	2.61
RT70 HC_0.1TiN_1000th_	1.65	2.59
RT70 HC_0.5TiN_1st_	1.60	2.57
RT70 HC_0.5TiN_500th_	1.59	2.52
RT70 HC_0.5TiN_1000th_	1.58	2.36
RT70 HC_1.0TiN_1st_	1.56	2.50
RT70 HC_1.0TiN_500th_	1.52	2.48
RT70 HC_1.0TiN_1000th_	1.52	2.26
RT70 HC_1.5TiN_1st_	1.52	2.32
RT70 HC_1.5TiN_500th_	1.44	2.22
RT70 HC_1.5TiN_1000th_	1.26	2.21
RT70 HC_2.0TiN_1st_	1.47	2.22
RT70 HC_2.0TiN_500th_	1.41	2.14
RT70 HC_2.0TiN_1000th_	1.26	2.01

**Table 5:** Tod, Tmd, and Rw values of the pristine RT70 HC and RT70 HC_TiN nanocomposite PCMs.

Sample Name	*T*_od_ (°C)	*T*_md_ (°C)	*Rw*
RT70 HC	240.4	273.7	1.68
RT70 HC_0.1TiN	244.6	279.8	2.91
RT70 HC_0.5TiN	240.8	273.4	4.14
RT70 HC_1.0TiN	247.1	272.5	6.04
RT70 HC_1.5TiN	248.0	280.0	7.33
RT70 HC_2.0TiN	244.3	279.6	9.60

*T_od_* = extrapolated onset degradation temperature, *T_md_* = temperature of maximum degradation rate (DTG peak), *Rw* = residual weight at 400 °C; because the residue remains constant between 400 and 500 °C (Figure 11), the values at 400 °C and 500 °C coincide.

**Table 6:** Characteristic FT-IR band positions of the RT70 HC_TiN nanocomposite PCMs before and after 1000 thermal cycles.

Band Assignment	Before Cycling (cm^−1^)	After 1000 Cycles (cm^−1^)
Asymmetric CH_2_/CH_3_ stretching	2915	2914
Symmetric CH_2_/CH_3_ stretching	2847	2847
CH_2_/CH_3_ bending	1467	1468
CH_2_ rocking	719	719

**Table 7:** Main properties of the RT70 HC and TiN used in this study. The RT70 HC values are taken from the Rubitherm Technologies GmbH datasheet, and the TiN supplier, purity, particle size, and crystal structure are taken from the Nanografi Nanotechnology datasheet. The density, melting point, thermal conductivity, and specific heat capacity of TiN are representative literature values for titanium nitride [33].

Material	Property	Value
RT70 HC	Supplier	Rubitherm Technologies GmbH, Germany
RT70 HC	Material class	Paraffin-based organic PCM
RT70 HC	Melting range	69–71 °C
RT70 HC	Peak melting temperature	~70 °C
RT70 HC	Congealing range	71–69 °C
RT70 HC	Heat storage capacity	260 kJ/kg
RT70 HC	Thermal conductivity	0.2 W/(m·K)
RT70 HC	Specific heat capacity	2 kJ/(kg·K)
RT70 HC	Solubility in water	Insoluble
RT70 HC	Appearance	White solid
TiN	Supplier	Nanografi, Türkiye
TiN	Material type	Titanium nitride nanopowder
TiN	Purity	99.2%+
TiN	Average particle size	20 nm
TiN	Crystal structure	Cubic
TiN	Thermophysical properties	ρ 5.22 g/cm^3^, Tm ~2930 °C, k ~19–29 W/(m·K), Cp ~0.60 J/(g·°C)

**Table 8:** Sample codes and composition ratios of the pristine RT70 HC and RT70 HC_TiN nanocomposite PCMs. SDS, added as a dispersing aid at a fixed 2:1 SDS:TiN mass ratio, is reported as an additional component and is not included in the 100 wt.% normalization (see Section 3.2).

Sample Code	RT70 HC (wt.%)	TiN (wt.%)	SDS (wt.%)
RT70 HC	100	0.0	0.0
RT70 HC_0.1TiN	99.9	0.1	0.2
RT70 HC_0.5TiN	99.5	0.5	1.0
RT70 HC_1.0TiN	99.0	1.0	2.0
RT70 HC_1.5TiN	98.5	1.5	3.0
RT70 HC_2.0TiN	98.0	2.0	4.0
RT70 HC_SDS	100	0.0	4.0

The RT70 HC_SDS sample is a surfactant-only control (pristine RT70 HC with 4.0 wt.% SDS and no TiN, matching the highest SDS level used in the composites). It was prepared to isolate the SDS contribution and was characterized in the 1st cycle only, so it was not included in the 1000-cycle program (see Section 2.5, Section 2.6 and Section 2.7).

## Data Availability

The data presented in this study are available on request from the corresponding author.
